# Contemporary EQ-5D-5​L reference values for adults in Scotland: a secondary analysis of the SIPHER-HWMIC survey

**DOI:** 10.1007/s11136-026-04433-3

**Published:** 2026-11-12

**Authors:** Ling Jie Cheng, Xun Li

**Affiliations:** 1 Alice Lee Centre for Nursing Studies, Centre for Translational Medicine, Yong Loo Lin School of Medicine, National University of Singapore, Block MD6, Level 5, 14 Medical Drive, Singapore, 117599 Singapore; 2 Nuffield Department of Women’s & Reproductive Health, National Perinatal Epidemiology Unit, University of Oxford, Level 2, New Radcliffe House, Radcliffe Observatory Quarter, Woodstock Road, Oxford, OX2 6GG UK; 3 School of Engineering, Computing, and Mathematics, Oxford Brookes University, Oxford, UK

**Keywords:** EQ-5D-5L, Population norms, Reference values, Health-related quality of life, Value set, Scotland

## Abstract

**Purpose:**

Contemporary EQ-5D-5​L reference values for Scotland are lacking. We estimated values for Scottish adults using the United Kingdom value set.

**Methods:**

We analysed the 2022 SIPHER-HWMIC online panel survey, which oversampled Scottish residents. We included adults with complete EQ-5D-5​L responses and valid EQ VAS data (n = 7,776). Index values were calculated using the Rowen et al. [27] United Kingdom value set, with the Devlin et al. [26] England value set as a comparator. Estimates were post-stratified to Scotland’s age-by-sex distribution using mid-2022 population counts. Sensitivity analyses assessed weighting, alternative value sets, and other specifications.

**Results:**

The weighted mean EQ-5D-5​L index was 0.827 (95% confidence interval [CI] 0.821–0.833), mean EQ VAS was 73.1 (95% CI 72.6–73.6), and 20.7% reported full health. Problems were most common in pain/discomfort (60.3%) and anxiety/depression (53.9%), and least common in self-care (14.9%). Anxiety/depression problems were highest among younger adults, particularly women aged 18–24 years (79.9%), whereas mobility, usual activities, and pain/discomfort problems generally increased with age. Index and EQ VAS scores were lowest among respondents who were long-term sick or disabled, and were also lower among those with lower incomes or substantial caring responsibilities. Weighting changed the mean index marginally (0.820–0.827); the Devlin et al. value set yielded 0.819. Findings were otherwise robust to sensitivity analyses.

**Conclusion:**

These contemporary reference values can support interpretation of EQ-5D-5​L data in Scottish clinical, public health, and economic evaluations. Given recruitment through non-probability online panels, they should be regarded as general-population reference values rather than nationally representative norms.

**Supplementary Information:**

The online version contains supplementary material available at https://doi.org/10.1007/s11136-026-04433-3.

## Introduction

The EQ-5D is one of the most widely used generic preference-based measures of health-related quality of life (HRQoL) [1, 2]. It comprises a descriptive system covering five dimensions: mobility, self-care, usual activities, pain/discomfort and anxiety/depression, together with a visual analogue scale (EQ VAS), on which respondents rate their health today from 0 (the worst health you can imagine) to 100 (the best health you can imagine) [1]. Responses to the descriptive system can be converted into a single index value using a country-specific value set, with full health anchored at 1 and dead at 0; negative values indicate health states valued as worse than dead. This index enables the calculation of quality-adjusted life years for economic evaluation [1]. These properties have made the EQ-5D central to health technology assessment, population health surveillance and the interpretation of patient-reported outcomes [1].

The five-level version of the EQ-5D (EQ-5D-5​L) was developed to improve sensitivity and reduce the ceiling effects associated with the original three-level version [3]. Systematic reviews indicate that the EQ-5D-5​L generally has lower ceiling effects and greater informativity, although evidence regarding its comparative responsiveness remains mixed [4–6]. The EQ-5D-5​L also identifies health problems more frequently, but generally at lower severity levels [7]. The relative weights assigned to dimensions and severity levels differ between EQ-5D-5​L and EQ-5D-3​L value sets [8]. Consequently, index values derived directly from EQ-5D-5​L value sets are generally preferred to those obtained through indirect mapping approaches.

Population reference values, sometimes described as population norms, provide benchmarks against which the HRQoL of specific patient groups can be compared. They can also support the estimation of expected or baseline utility values in economic models and help quantify the burden of disease [9, 10]. EQ-5D-5​L reference values have been reported for numerous populations, including England [11], the United States [12], Norway [13], Germany [14, 15], Italy [16], Hungary [17], Ireland [18], Poland [19], Spain [20], Bulgaria [21], Moscow, Russia [22], Peninsular Malaysia [23] and Singapore [24]. Across these studies, physical health generally declines with age, and women tend to report more health problems than men. By contrast, anxiety and depression are more prevalent among younger adults in several populations [9, 13, 16, 17]. These patterns are the main points of comparison for the present study, and they show why reference values are most informative when reported by age, sex and dimension rather than as a single population mean.

For the United Kingdom, the choice of value set is an additional consideration. Before a directly elicited United Kingdom EQ-5D-5​L value set became available, EQ-5D-5​L responses could be scored by mapping them onto an EQ-5D-3​L value set [25] or by applying the directly elicited England value set developed by Devlin et al. [26]. A new value set based on preferences elicited from a representative sample of the United Kingdom public has now been published and is suitable for informing policy [27]. The National Institute for Health and Care Excellence has since adopted the new United Kingdom EQ-5D-5L value set as its preferred approach for adult reference-case analyses, subject to transitional arrangements [28]. The availability of this value set provides a strong rationale for producing contemporary EQ-5D-5​L reference values scored using United Kingdom population preferences.

Contemporary Scotland-specific EQ-5D-5​L reference values are lacking. This is important because Scotland has a distinct population health profile within the United Kingdom. Life expectancy remains approximately two years lower in Scotland than in England for both women and men [29], and substantial proportions of Scottish adults report long-term conditions, activity limitations and poor mental health [30]. Reference values derived from England or the wider United Kingdom may therefore not fully reflect the distribution of health in Scotland and may provide imperfect benchmarks for interpreting outcomes among Scottish patient groups. Scotland-specific reference values are needed to provide an appropriate general-population comparator and to support benchmarking using the newly published United Kingdom EQ-5D-5​L value set [27].

The Systems Science in Public Health and Health Economics Research (SIPHER) Health and Wellbeing Multi-Instrument Comparison (HWMIC) survey collected HRQoL and sociodemographic information from more than 12,000 adults in the United Kingdom in 2022 and deliberately oversampled respondents living in Scotland [31]. It therefore provides a timely opportunity to estimate Scotland-specific reference values. However, participants were recruited through commercial online panels rather than probability sampling. We therefore describe the resulting estimates as “general-population reference values” rather than “nationally representative population norms”. Although post-stratification weighting was used to improve alignment with the age-by-sex distribution of the Scottish adult population, residual selection bias may remain [32].

The primary objective of this study was to estimate contemporary EQ-5D-5​L reference values for adults in Scotland. Specifically, we aimed to describe the prevalence and severity of problems in each EQ-5D-5​L dimension; estimate EQ-5D-5​L index and EQ VAS values for the overall Scottish sample and by age and sex; examine variation in these outcomes across selected sociodemographic and health-related characteristics, thereby extending the reference values beyond age and sex and identifying characteristics associated with poorer HRQoL; and assess the robustness of the estimates to weighting, alternative value sets and other analytical specifications.

## Methods

### Study design, data source and sample

This secondary cross-sectional analysis used data from the SIPHER-HWMIC survey, obtained through the UK Data Service (study number 9458) [31]. The survey recruited adults aged 18 years or older through three commercial online panels in the United Kingdom in 2022 and deliberately oversampled residents of Scotland. It collected information on the EQ-5D-5​L descriptive system, EQ VAS and sociodemographic characteristics.

Of the 12,401 respondents in the full United Kingdom dataset, 7,776 were resident in Scotland. All had a complete EQ-5D-5​L profile and a non-missing EQ VAS response and were included in the analytic sample (age range 18–97 years). Sex used for weighting was derived from a self-reported gender item.

### EQ-5D-5​L measures, outcomes and value sets

Each EQ-5D-5​L dimension has five response levels: no problems, slight problems, moderate problems, severe problems, and extreme problems or unable to, depending on the dimension. Responses form a five-digit health-state profile ranging from 11​111, indicating no problems in any dimension, to 55​555, indicating the most severe level in every dimension, giving 3,125 (5^5^) possible health states.

We examined the distribution of responses across the five levels of each dimension; the prevalence of any problems (levels 2–5) and moderate-to-extreme problems (levels 3–5); the prevalence of full health (state 11​111); the frequencies of individual health states; the level sum score, calculated by summing the five dimension levels and ranging from 5 to 25; the number of dimensions with any problems; the EQ-5D-5​L index; and the EQ VAS.

The primary EQ-5D-5​L index was calculated using the Rowen et al. [27] United Kingdom value set, which has a possible range of − 0.567 to 1. The Devlin et al. [26] England value set was used as a comparator. We implemented both additive scoring algorithms using the published dimension-level coefficients and verified the calculations against published worked examples. We did not use the van Hout et al. [25] crosswalk, which maps EQ-5D-5​L responses onto EQ-5D-3​L-based index values.

### Post-stratification weighting

Post-stratification weights were constructed using National Records of Scotland mid-2022 population estimates [33] to align the sample with the joint age-by-sex distribution of adults aged 18 years or older in Scotland. We used seven age bands: 18–24, 25–34, 35–44, 45–54, 55–64, 65–74 and 75 years or older. Weights were calculated for the 7,747 respondents recorded as male or female and normalised to a mean of 1. The weights ranged from 0.56 to 5.79. We quantified the loss of precision attributable to unequal weighting using the unequal-weighting design effect, calculated as $n\sum {w}_{i}^{2}/(\sum {w}_{i}{)}^{2}$, and calculated the effective sample size as the weighted analytic sample size divided by the design effect.

The 29 respondents recorded as “other” could not be matched to a corresponding population target and were therefore excluded from weighted analyses but retained in unweighted analyses. Because the weight was constant within each joint age-by-sex cell, weighting did not affect cell-specific estimates. Age-by-sex reference values were therefore reported unweighted, whereas overall and sociodemographic subgroup estimates were reported primarily using weighted analyses, with unweighted estimates provided for comparison. The dataset did not contain sufficiently detailed geographical information to adjust for the Scottish Index of Multiple Deprivation, health board or urban–rural classification.

### Statistical analysis

Participant characteristics were summarised using frequencies and percentages for categorical variables and means with standard deviations, medians with interquartile ranges, and percentiles for continuous variables. Reference values were estimated overall, by age band and sex, and across subgroups defined by education, employment status, disposable household income after housing costs, total personal income, ethnicity, responsibility for dependent children, caring responsibilities and household size.

We reported 95% confidence intervals (CIs) throughout. Taylor-series linearisation was used for weighted estimates, Wilson score intervals for unweighted proportions and *t*-based intervals for unweighted means. CIs for proportions were constrained to the range of 0–100%. For subgroups with an unweighted sample size below 30, the sample size was retained for transparency, but outcome estimates were not reported because they were considered unstable.

As a secondary explanatory analysis, we fitted separate unweighted multivariable ordinary least squares models for the EQ-5D-5​L index and EQ VAS, using heteroskedasticity-robust standard errors. Covariates included age band, sex, education (degree or below degree), employment status (employed, retired, long-term sick or disabled, or unemployed or otherwise inactive), monthly disposable household income after housing costs (≤ £1040, £1041–£2080 or > £2080), ethnicity (White or non-White), caring responsibilities (none, < 20 or ≥ 20 h per week), responsibility for dependent children and recruitment-panel fixed effects. “Prefer not to answer” responses were retained as separate categories. Analyses were restricted to respondents recorded as male or female (n = 7,747). Coefficients were interpreted as associations rather than causal effects.

The principal robustness comparisons examined weighted versus unweighted estimates and index values calculated using the Rowen et al. [27] United Kingdom value set versus the Devlin et al. [26] England value set. Additional sensitivity analyses of the headline reference values used weights trimmed at the 1st and 99th percentiles; an alternative weighting scheme with four broader age bands (18–34, 35–54, 55–74 and 75 years or older), each by sex, with National Records of Scotland targets aggregated to these bands; exclusion of respondents with completion times below 6 min; exclusion of respondents with completion times below one-third of the median; and estimates calculated separately for each recruitment panel after standardisation to the Scottish age-by-sex distribution. We also examined post hoc whether the age pattern of anxiety/depression problems persisted within the largest recruitment panel and after excluding rapid completions, and whether it was mirrored by three other mental-health items collected in the same survey: the EQ Health and Wellbeing Short (EQ-HWB-S) items on feeling anxious and on feeling sad or depressed in the last 7 days, and the anxiety item of the Office for National Statistics personal well-being questions (ONS-4), which asks how anxious respondents felt yesterday.

Sensitivity analyses of the regression models comprised a Tobit model right-censored at an EQ-5D-5​L index value of 1 and weighted ordinary least squares models for the EQ-5D-5​L index and EQ VAS. These models used the same analytic sample and covariates as the primary models. For each weighted sensitivity analysis, post-stratification weights were recalculated within the relevant analysis sample. All statistical tests were two-sided, and *p* < 0.05 was considered statistically significant. Analyses followed a prespecified statistical analysis plan and were conducted using Stata version 19.0 (StataCorp, College Station, TX, USA).

## Results

### Sample characteristics

The analytic sample comprised 7,776 adults living in Scotland (Table 1). Compared with mid-2022 Scottish population estimates, the unweighted sample over-represented adults aged 55–64 years (26.4% versus 17.6%) and under-represented those aged 18–24 years (4.0% versus 10.5%). Respondents recorded as female were also over-represented (58.4% versus 52.0%). Most respondents were White (97.8%); 35.9% had a university degree, 37.9% were in full-time employment and 31.0% were retired. Most respondents were recruited through STV (94.7%), followed by MRFGR (3.5%) and Prolific (1.7%) (Table 1); in the weighted analyses, these panels comprised 7,339, 274 and 134 respondents, respectively. Post-stratification reproduced the target joint age-by-sex distribution, with a design effect of approximately 1.46 and an effective sample size of approximately 5290.

**Table 1:** Characteristics of the Scottish analytic sample

Characteristic	n	Unweighted, %	Weighted, %^a^	NRS 2022, %^b^
*Age band*				
18–24	309	4.0	10.5	10.5
25–34	826	10.6	15.7	15.7
35–44	978	12.6	15.4	15.4
45–54	1,412	18.2	16.1	16.1
55–64	2,049	26.4	17.6	17.6
65–74	1,709	22.0	13.5	13.5
75 +	493	6.3	11.2	11.2
*Sex*				
Male	3,205	41.2	48.0	48.0
Female	4,542	58.4	52.0	52.0
Other	29	0.4	–	–
*Recruitment panel*^c^				
STV	7,366	94.7	92.9	–
MRFGR	275	3.5	4.5	–
Prolific	135	1.7	2.6	–
*Highest educational qualification*				
No formal qualification	520	6.7	5.9	–
GCSE or equivalent	1,297	16.7	16.1	–
A-level or equivalent	1,491	19.2	20.6	–
University degree (undergraduate or postgraduate)	2,790	35.9	36.8	–
Other qualification	1,540	19.8	18.9	–
Prefer not to answer	138	1.8	1.7	–
*Ethnicity*				
White	7,603	97.8	97.4	–
Black, Black British, Caribbean or African	16	0.2	0.3	–
Asian/Asian British	37	0.5	0.6	–
Mixed or multiple ethnic groups	39	0.5	0.7	–
Other ethnic group	12	0.2	0.2	–
Prefer not to answer	69	0.9	0.8	–
*Employment status*				
Full-time (employed/self-employed)	2,944	37.9	42.8	–
Part-time (employed/self-employed)	1,108	14.2	12.7	–
Job-seeking	112	1.4	1.8	–
Long-term sick or disabled	563	7.2	6.6	–
Carer	141	1.8	1.6	–
Looking after home/family	183	2.4	2.0	–
Retired	2,413	31.0	26.4	–
Student	132	1.7	3.7	–
Other	121	1.6	1.5	–
Prefer not to answer	59	0.8	0.8	–
*Disposable household income after housing costs (monthly)*				
≤ £690	2,014	25.9	25.4	–
£691–£1,040	1,187	15.3	15.6	–
£1,041–£1,380	796	10.2	11.0	–
£1,381–£1,730	609	7.8	7.6	–
£1,731–£2,080	590	7.6	7.3	–
> £2,080	1,131	14.5	14.7	–
Prefer not to answer	1,449	18.6	18.4	–
*Total personal income before deductions, including benefits (annual)*				
No income	407	5.2	5.4	–
≤ £10,800	1,206	15.5	15.0	–
£10,801–£14,400	907	11.7	11.0	–
£14,401–£19,200	792	10.2	9.9	–
£19,201–£26,400	1,142	14.7	14.8	–
£26,401–£37,200	949	12.2	13.3	–
£37,201–£52,800	655	8.4	9.3	–
£52,801–£74,000	240	3.1	3.4	–
> £74,000	148	1.9	2.0	–
Prefer not to answer	1,330	17.1	16.0	–
*Responsibility for dependent children aged < 18 years*				
Yes	1,377	17.7	19.5	–
No	6,376	82.0	80.0	–
Prefer not to answer	23	0.3	0.4	–
*Caring for someone with a long-term illness or old-age needs*				
No	6,048	77.8	80.4	–
Yes, ≤ 9 h/week	716	9.2	8.2	–
Yes, 10–19 h/week	222	2.9	2.4	–
Yes, 20–34 h/week	133	1.7	1.5	–
Yes, 35–49 h/week	146	1.9	1.6	–
Yes, ≥ 50 h/week	396	5.1	4.4	–
Prefer not to answer	115	1.5	1.5	–

n and unweighted percentages include all Scottish respondents (n = 7,776); GCSE General Certificate of Secondary Education, NRS National Records of Scotland

^a^Weighted percentages use the male/female analytic base (n = 7,747); 29 respondents recorded as “Other” sex could not be assigned a National Records of Scotland weight. The sex categories used for weighting were mapped from the survey gender variable

^b^Post-stratification target: the joint age-band-by-sex distribution of Scottish adults aged 18 years or older (National Records of Scotland, mid-2022)

^c^Recruitment panels: STV is a Scottish panel provider; MRFGR and Prolific are UK panel providers. In the weighted analyses (n = 7,747), the panels comprised 7339, 274 and 134 respondents, respectively

### Distribution of responses across dimensions

The weighted proportion reporting no problems was highest for self-care (85.1%) and mobility (68.8%) and lowest for pain/discomfort (39.7%) and anxiety/depression (46.1%) (Table 2). The prevalence of any problems (levels 2–5) was highest for pain/discomfort (60.3%, 95% CI 58.9 to 61.6) and anxiety/depression (53.9%, 95% CI 52.6 to 55.3), followed by usual activities (34.5%) and mobility (31.2%), and was lowest for self-care (14.9%) (Table 3). Moderate-to-extreme problems (levels 3–5) were most prevalent for anxiety/depression (24.6%) and pain/discomfort (21.3%) and least prevalent for self-care (6.2%).

**Table 2:** Weighted distribution of EQ-5D-5​L responses across severity levels, by dimension

Dimension	1 No problems	2 Slight	3 Moderate	4 Severe	5 Extreme/unable
Mobility	68.8 (67.6, 70.0)	17.8 (16.9, 18.9)	8.2 (7.6, 8.9)	4.5 (4.0, 5.0)	0.6 (0.4, 0.8)
Self-care	85.1 (84.1, 86.0)	8.7 (7.9, 9.4)	4.6 (4.0, 5.1)	1.3 (1.1, 1.6)	0.3 (0.2, 0.5)
Usual activities	65.5 (64.2, 66.7)	20.8 (19.7, 21.9)	8.9 (8.2, 9.7)	3.9 (3.4, 4.4)	1.0 (0.7, 1.3)
Pain/discomfort	39.7 (38.4, 41.1)	39.0 (37.7, 40.3)	15.0 (14.1, 15.9)	5.0 (4.5, 5.6)	1.3 (1.1, 1.6)
Anxiety/depression	46.1 (44.7, 47.4)	29.4 (28.1, 30.6)	16.5 (15.5, 17.5)	5.7 (5.0, 6.4)	2.4 (2.1, 2.9)

Cells show weighted percentage (95% confidence interval); percentages sum to 100% within each dimension, subject to rounding. Weighted analytic base: n = 7,747. Confidence intervals use linearised standard errors and are bounded on the percentage scale

**Table 3:** Prevalence of EQ-5D-5​L problems by age band and sex

Age band	n	Mobility	Self-care	Usual activities	Pain/discomfort	Anxiety/depression
*Panel A*: * Any problems (levels 2–5), % (95% confidence interval)*						
*Males*						
18–24	70	17.1 (10.1, 27.6)	14.3 (7.9, 24.3)	27.1 (18.1, 38.5)	38.6 (28.0, 50.3)	65.7 (54.0, 75.8)
25–34	250	11.6 (8.2, 16.2)	8.0 (5.2, 12.0)	18.8 (14.4, 24.1)	40.8 (34.9, 47.0)	60.4 (54.2, 66.3)
35–44	315	21.9 (17.7, 26.8)	10.8 (7.8, 14.7)	26.7 (22.1, 31.8)	59.0 (53.5, 64.3)	61.6 (56.1, 66.8)
45–54	503	28.0 (24.3, 32.1)	11.3 (8.8, 14.4)	28.2 (24.5, 32.3)	57.5 (53.1, 61.7)	50.5 (46.1, 54.8)
55–64	861	38.3 (35.1, 41.6)	16.0 (13.7, 18.6)	35.5 (32.4, 38.8)	67.4 (64.2, 70.4)	45.1 (41.8, 48.4)
65–74	898	46.4 (43.2, 49.7)	19.4 (16.9, 22.1)	41.3 (38.1, 44.6)	74.1 (71.1, 76.8)	35.5 (32.5, 38.7)
75 +	308	51.9 (46.4, 57.5)	16.6 (12.8, 21.1)	50.0 (44.5, 55.5)	76.0 (70.9, 80.4)	30.2 (25.3, 35.5)
*Females*						
18–24	229	16.6 (12.3, 22.0)	20.1 (15.4, 25.8)	35.4 (29.5, 41.8)	46.3 (39.9, 52.8)	79.9 (74.2, 84.6)
25–34	567	12.5 (10.0, 15.5)	10.6 (8.3, 13.4)	26.3 (22.8, 30.1)	43.4 (39.4, 47.5)	66.1 (62.1, 69.9)
35–44	660	22.0 (19.0, 25.3)	12.3 (10.0, 15.0)	28.3 (25.0, 31.9)	56.7 (52.9, 60.4)	69.1 (65.5, 72.5)
45–54	906	34.4 (31.4, 37.6)	16.6 (14.3, 19.1)	37.0 (33.9, 40.2)	66.0 (62.9, 69.0)	58.4 (55.2, 61.6)
55–64	1,185	41.9 (39.2, 44.8)	19.2 (17.0, 21.5)	41.9 (39.1, 44.7)	70.9 (68.2, 73.4)	51.8 (49.0, 54.6)
65–74	810	46.3 (42.9, 49.7)	18.4 (15.9, 21.2)	46.8 (43.4, 50.2)	71.0 (67.8, 74.0)	43.0 (39.6, 46.4)
75 +	185	50.8 (43.7, 57.9)	17.8 (13.0, 24.0)	47.0 (40.0, 54.2)	74.1 (67.3, 79.8)	34.1 (27.6, 41.1)
*All ages*						
Unweighted	7,776	34.7 (33.7, 35.8)	16.0 (15.2, 16.8)	36.8 (35.7, 37.8)	64.0 (62.9, 65.1)	51.9 (50.8, 53.0)
Weightedᵃ	7,747	31.2 (30.0, 32.4)	14.9 (14.0, 15.9)	34.5 (33.3, 35.8)	60.3 (58.9, 61.6)	53.9 (52.6, 55.3)
*Panel B*: *Moderate-to-extreme problems (levels 3–5), % (95% confidence interval)*						
*Males*						
18–24	70	7.1 (3.1, 15.7)	4.3 (1.5, 11.9)	15.7 (9.0, 26.0)	12.9 (6.9, 22.7)	34.3 (24.2, 46.0)
25–34	250	3.2 (1.6, 6.2)	4.8 (2.8, 8.2)	5.2 (3.1, 8.7)	5.2 (3.1, 8.7)	26.8 (21.7, 32.6)
35–44	315	8.3 (5.7, 11.8)	4.8 (2.9, 7.7)	10.5 (7.6, 14.3)	14.6 (11.1, 18.9)	30.8 (26.0, 36.1)
45–54	503	8.9 (6.8, 11.8)	5.4 (3.7, 7.7)	9.9 (7.6, 12.9)	17.9 (14.8, 21.5)	23.1 (19.6, 26.9)
55–64	861	17.9 (15.5, 20.6)	8.0 (6.4, 10.0)	15.1 (12.9, 17.6)	24.7 (22.0, 27.7)	19.2 (16.7, 21.9)
65–74	898	20.4 (17.9, 23.1)	8.1 (6.5, 10.1)	16.4 (14.1, 18.9)	27.7 (24.9, 30.7)	13.9 (11.8, 16.3)
75+	308	23.4 (19.0, 28.4)	6.5 (4.2, 9.8)	18.8 (14.9, 23.6)	27.9 (23.2, 33.2)	11.0 (8.0, 15.0)
*Females*						
18–24	229	3.1 (1.5, 6.2)	5.2 (3.0, 8.9)	9.6 (6.4, 14.1)	13.1 (9.3, 18.1)	40.6 (34.5, 47.1)
25–34	567	3.9 (2.6, 5.8)	3.9 (2.6, 5.8)	8.5 (6.4, 11.0)	13.2 (10.7, 16.3)	34.0 (30.3, 38.0)
35–44	660	7.7 (5.9, 10.0)	3.8 (2.6, 5.5)	10.5 (8.3, 13.0)	18.3 (15.6, 21.5)	31.8 (28.4, 35.5)
45–54	906	16.9 (14.6, 19.5)	7.9 (6.4, 9.9)	17.2 (14.9, 19.8)	26.9 (24.1, 29.9)	27.4 (24.6, 30.4)
55–64	1,185	20.4 (18.2, 22.8)	8.9 (7.5, 10.7)	18.7 (16.6, 21.1)	32.1 (29.5, 34.8)	22.1 (19.8, 24.6)
65–74	810	23.2 (20.4, 26.2)	7.7 (6.0, 9.7)	19.6 (17.0, 22.5)	33.7 (30.5, 37.0)	16.0 (13.7, 18.7)
75+	185	22.7 (17.3, 29.3)	7.0 (4.2, 11.7)	18.9 (13.9, 25.2)	27.6 (21.6, 34.4)	11.9 (8.0, 17.3)
*All ages*						
Unweighted	7,776	15.5 (14.7, 16.3)	6.9 (6.4, 7.5)	14.9 (14.2, 15.8)	24.3 (23.3, 25.2)	23.2 (22.2, 24.1)
Weighted^a^	7,747	13.3 (12.5, 14.2)	6.2 (5.6, 6.9)	13.8 (12.9, 14.7)	21.3 (20.3, 22.3)	24.6 (23.4, 25.8)

Age-band-by-sex cells show unweighted percentages with Wilson score confidence intervals. Because the post-stratification weight is constant within each joint age-band-by-sex cell, weighting does not change these cell-specific point estimates

^a^Weighted by post-stratification to the National Records of Scotland mid-2022 age-band-by-sex distribution; weighted confidence intervals use linearised standard errors and are bounded on the percentage scale

### Problems by age and sex

Problems with mobility, usual activities and pain/discomfort generally became more prevalent with age in both sexes. Anxiety/depression showed the opposite age pattern, with the highest prevalence among the youngest adults and a decline with increasing age (Table 3). Anxiety/depression problems were particularly common among women aged 18–24 years, of whom 79.9% reported any problems and 40.6% reported moderate-to-extreme problems. Across most age bands and dimensions, women reported a higher prevalence of problems than men.

### EQ-5D-5​L index and EQ VAS reference values

The overall weighted mean EQ-5D-5​L index was 0.827 (95% CI 0.821 to 0.833; standard deviation 0.231; median 0.903), compared with an unweighted mean of 0.820 (95% CI 0.814 to 0.825) (Table 4). Full health (state 11​111) was reported by 20.7% of respondents in the weighted analysis and 20.0% in the unweighted analysis. The overall weighted mean EQ VAS was 73.1 (95% CI 72.6 to 73.6; median 80), compared with an unweighted mean of 72.6 (Table 5).

**Table 4:** EQ-5D-5​L index reference values by age band and sex

Age band	n	Mean (SD)	95% CI	Median (IQR)	Full healthᵃ, % (95% CI)
*Males*					
18–24	70	0.848 (0.196)	(0.802, 0.895)	0.908 (0.818, 1.000)	27.1 (18.1, 38.5)
25–34	250	0.879 (0.162)	(0.859, 0.900)	0.944 (0.853, 1.000)	28.4 (23.2, 34.3)
35–44	315	0.828 (0.233)	(0.802, 0.853)	0.903 (0.790, 0.959)	21.0 (16.8, 25.8)
45–54	503	0.847 (0.220)	(0.828, 0.867)	0.912 (0.818, 1.000)	25.0 (21.5, 29.0)
55–64	861	0.825 (0.250)	(0.808, 0.842)	0.910 (0.812, 0.959)	21.7 (19.1, 24.6)
65–74	898	0.828 (0.232)	(0.813, 0.843)	0.903 (0.812, 0.944)	19.5 (17.0, 22.2)
75 +	308	0.830 (0.205)	(0.807, 0.853)	0.895 (0.800, 0.944)	14.9 (11.4, 19.3)
*Females*					
18–24	229	0.811 (0.198)	(0.785, 0.836)	0.874 (0.690, 0.959)	14.0 (10.1, 19.1)
25–34	567	0.849 (0.198)	(0.833, 0.866)	0.903 (0.818, 0.959)	24.0 (20.7, 27.7)
35–44	660	0.831 (0.220)	(0.814, 0.848)	0.903 (0.812, 0.959)	16.1 (13.5, 19.1)
45–54	906	0.790 (0.284)	(0.772, 0.809)	0.903 (0.785, 0.959)	17.3 (15.0, 19.9)
55–64	1,185	0.796 (0.271)	(0.780, 0.811)	0.895 (0.769, 0.959)	19.2 (17.0, 21.5)
65–74	810	0.807 (0.252)	(0.789, 0.824)	0.895 (0.786, 0.959)	21.4 (18.7, 24.3)
75 +	185	0.820 (0.225)	(0.787, 0.852)	0.902 (0.807, 0.944)	17.8 (13.0, 24.0)
*Both sexes (weighted)*^b^					
18–24	299	0.829 (0.197)	(0.803, 0.856)	0.903 (0.747, 0.959)	20.5 (15.4, 26.9)
25–34	817	0.864 (0.182)	(0.851, 0.877)	0.910 (0.818, 1.000)	26.1 (23.0, 29.6)
35–44	975	0.829 (0.226)	(0.814, 0.844)	0.903 (0.812, 0.959)	18.4 (16.0, 21.2)
45–54	1,409	0.818 (0.256)	(0.805, 0.831)	0.903 (0.812, 0.959)	21.1 (18.9, 23.4)
55–64	2,046	0.810 (0.261)	(0.799, 0.821)	0.903 (0.786, 0.959)	20.4 (18.7, 22.2)
65–74	1,708	0.817 (0.243)	(0.805, 0.828)	0.903 (0.790, 0.944)	20.5 (18.6, 22.5)
75 +	493	0.824 (0.217)	(0.803, 0.845)	0.902 (0.800, 0.944)	16.6 (13.3, 20.5)
*All ages*					
Unweighted	7,776	0.820 (0.242)	(0.814, 0.825)	0.903 (0.796, 0.959)	20.0 (19.1, 20.9)
Weighted	7,747	0.827 (0.231)	(0.821, 0.833)	0.903 (0.808, 0.959)	20.7 (19.6, 21.8)

The EQ-5D-5​L index was calculated using the Rowen et al. [27] UK value set (range − 0.567 to 1). Age-band-by-sex estimates are unweighted and weight-invariant; all-age estimates are shown unweighted and weighted

^a^Full health: EQ-5D-5​L health state 11​111 (index = 1). CI confidence interval, IQR interquartile range, SD standard deviation

^b^Both sexes combined, weighted to the National Records of Scotland mid-2022 age-band-by-sex distribution (male/female base, n = 7,747). Combining the sexes within an age band is not weight-invariant, so these rows are weighted

**Table 5:** EQ VAS reference values by age band and sex

Age band	n	Mean (SD)	95% CI	P10	P25	Median	P75	P90
*Males*								
18–24	70	76.7 (18.4)	(72.3, 81.1)	50	70	80	90	94
25–34	250	76.1 (17.0)	(74.0, 78.2)	55	70	80	90	95
35–44	315	70.2 (19.8)	(68.0, 72.4)	40	60	75	85	90
45–54	503	72.2 (20.1)	(70.5, 74.0)	40	65	80	85	90
55–64	861	73.3 (20.7)	(71.9, 74.7)	40	65	80	90	95
65–74	898	72.8 (20.8)	(71.4, 74.1)	40	60	80	90	95
75 +	308	73.4 (20.1)	(71.2, 75.7)	40	60	80	90	95
*Females*								
18–24	229	73.9 (20.2)	(71.3, 76.6)	45	65	80	90	95
25–34	567	74.5 (18.3)	(73.0, 76.0)	50	65	80	90	95
35–44	660	73.1 (19.8)	(71.5, 74.6)	48	65	80	90	95
45–54	906	70.2 (22.3)	(68.8, 71.7)	35	60	76	89	95
55–64	1,185	71.5 (22.1)	(70.3, 72.8)	40	60	80	90	95
65–74	810	72.8 (21.0)	(71.4, 74.3)	40	60	80	90	95
75 +	185	74.4 (19.4)	(71.6, 77.2)	50	65	80	90	90
*Both sexes (weighted)*^a^								
18–24	299	75.3 (19.3)	(72.8, 77.8)	50	70	80	90	95
25–34	817	75.3 (17.6)	(74.0, 76.6)	50	70	80	90	95
35–44	975	71.7 (19.8)	(70.4, 73.0)	40	60	75	85	92
45–54	1,409	71.2 (21.3)	(70.1, 72.3)	40	60	80	88	92
55–64	2,046	72.4 (21.4)	(71.5, 73.3)	40	60	80	90	95
65–74	1,708	72.8 (20.9)	(71.8, 73.8)	40	60	80	90	95
75 +	493	74.0 (19.7)	(72.1, 75.9)	50	60	80	90	93
*All ages*								
Unweighted	7,776	72.6 (20.7)	(72.1, 73.0)	40	60	80	90	95
Weighted	7,747	73.1 (20.2)	(72.6, 73.6)	40	62	80	90	95

EQ VAS measures self-rated health today from 0 to 100, with higher scores indicating better health. Age-band-by-sex estimates are unweighted and weight-invariant; all-age estimates are shown unweighted and weighted

CI confidence interval, P10–P90 10th to 90th percentiles, SD standard deviation

^a^Both sexes combined, weighted to the National Records of Scotland mid-2022 age-band-by-sex distribution (male/female base, n = 7,747). Combining the sexes within an age band is not weight-invariant, so these rows are weighted. Weighted percentiles are shown

**Fig. 1:**
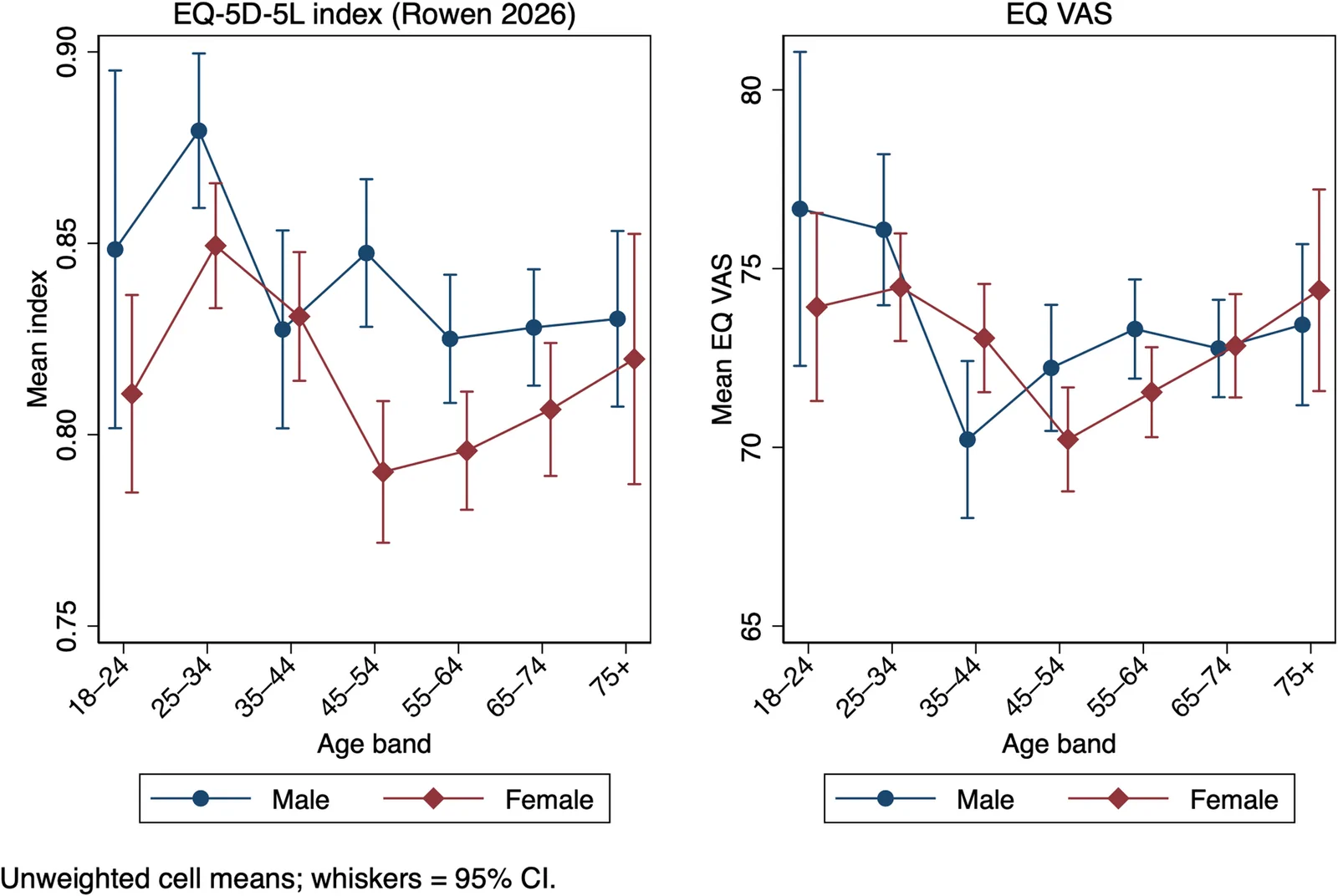
Mean EQ-5D-5L index (left panel) and EQ VAS (right panel), with 95% confidence intervals, by age band and sex. Blue circles denote males and red diamonds denote females. Age-band-by-sex estimates are unweighted and weight-invariant because the post-stratification weight is constant within each joint age-band-by-sex cell. The vertical axes do not begin at zero

Age-by-sex reference values are presented in Tables 4 and 5 and Fig. 1. With both sexes combined, the weighted mean index ranged from 0.810 at 55–64 years to 0.864 at 25–34 years and did not decline consistently with age. Differences between age-by-sex groups were larger, with means ranging from 0.790 in women aged 45–54 years to 0.879 in men aged 25–34 years. Among adults aged 18–24 years, the mean index was lower in women than in men (0.811 versus 0.848). Mean EQ VAS scores ranged from 70.2 to 76.7 across age-by-sex groups and from 71.2 to 75.3 across age bands with both sexes combined.

### Sociodemographic subgroups

In descriptive subgroup analyses, EQ-5D-5​L index values, EQ VAS scores and the proportion reporting full health were generally lower among respondents with lower educational attainment, lower household or personal income, economic inactivity, greater caring responsibilities and those living alone (Table S1). The lowest values were observed among respondents classified as long-term sick or disabled, whose weighted mean index was 0.389, mean EQ VAS was 43.0 and prevalence of full health was 0.5%. Values were also particularly low among respondents providing 20 or more hours of care per week, while both the mean index and mean EQ VAS generally increased with income.

Income questions were declined far more often than other items: 18.6% of respondents preferred not to report disposable household income and 17.1% total personal income (Table 1), and 13.4% declined both. In a post hoc comparison, respondents who declined to report household income were older (31.8% versus 23.1% aged 65 years or older), more often female (59.9% versus 50.3%), retired (32.2% versus 25.1%) or long-term sick or disabled (9.2% versus 6.1%), and less often held a degree (29.3% versus 38.6%) than those who answered. Their weighted mean index was lower (0.802 versus 0.833; difference after adjustment for age band and sex − 0.026, 95% CI − 0.043 to − 0.009), whereas their mean EQ VAS was similar (72.4 versus 73.2).

### Health states, level sum score and value sets

Of the 3,125 possible health states, 594 (19.0%) were reported, 284 of them by a single respondent. The most frequently reported health state was full health (11​111), reported by 20.7% of respondents in the weighted analysis, followed by 11​121 (10.6%), 11​112 (9.6%) and 11​122 (7.1%) (Table S2). These states represent isolated or combined slight problems with pain/discomfort and anxiety/depression, with no problems in the other dimensions.

The weighted mean level sum score was 8.06 (95% CI 7.97 to 8.14) (Table S3), and 20.7% of respondents reported no problems in any dimension (Table S4). The weighted mean index was similar when calculated using the Rowen et al. United Kingdom value set [27] and the Devlin et al. England value set [26]: 0.827 and 0.819, respectively. However, the corresponding medians differed more noticeably, at 0.903 and 0.872, respectively (Table S5), and the distributions differed in shape (Figure S1). Values were higher under the Rowen et al. value set at the 25th percentile (0.808 versus 0.758) and 75th percentile (0.959 versus 0.937) but lower at the 10th percentile (0.550 versus 0.581), and 1.7% versus 1.0% of respondents had values below 0. Because index values cluster on a few frequently reported health states, medians depend on how those states are valued; for example, state 11112 is valued at 0.959 under the Rowen et al. value set and 0.922 under the Devlin et al. value set, and state 11​122 at 0.903 and 0.859, respectively (Table S2).

### Factors associated with the index and EQ VAS

In the multivariable models, being classified as long-term sick or disabled had the largest association with both outcomes (index coefficient − 0.489, 95% CI − 0.519 to − 0.458; EQ VAS coefficient − 32.5, 95% CI − 34.4 to − 30.6) (Table 6). Lower household income was also associated with poorer outcomes. Compared with the highest income category, the lowest category was associated with an index coefficient of − 0.060 and an EQ VAS coefficient of − 5.8. Providing at least 20 h of care per week was associated with an index coefficient of − 0.053 and an EQ VAS coefficient of − 4.5. Unemployment or other economic inactivity and retirement were also associated with lower scores.

**Table 6:** Factors associated with the EQ-5D-5L index and EQ VAS: unweighted multivariable ordinary least squares models

Characteristic	EQ-5D-5​L index, β (95% CI)	EQ VAS, β (95% CI)
*Age band (reference: 45–54)*		
18–24	0.010 (− 0.015, 0.036)	3.8 (1.3, 6.3)**
25–34	0.021 (0.006, 0.036)**	2.2 (0.7, 3.7)**
35–44	0.004 (− 0.012, 0.020)	0.2 (− 1.3, 1.7)
55–64	0.018 (0.002, 0.033)*	2.5 (1.2, 3.9)***
65–74	− 0.006 (− 0.025, 0.014)	0.5 (− 1.3, 2.3)
75 +	0.000 (− 0.027, 0.026)	1.3 (− 1.2, 3.7)
*Sex (reference: male)*		
Female	− 0.014 (− 0.023, − 0.004)**	0.4 (− 0.5, 1.3)
*Highest qualification (reference: degree)*		
Below degree	− 0.011 (− 0.020, − 0.002)*	− 0.3 (− 1.2, 0.6)
Prefer not to answer	− 0.045 (− 0.093, 0.003)	− 4.6 (− 8.3, − 0.9)*
*Employment (reference: employed)*		
Retired	− 0.033 (− 0.048, − 0.018)***	− 1.5 (− 2.9, − 0.1)*
Long-term sick or disabled	− 0.489 (− 0.519, − 0.458)***	− 32.5 (− 34.4, − 30.6)***
Unemployed or other inactive	− 0.049 (− 0.067, − 0.031)***	− 4.1 (− 5.8, − 2.3)***
Prefer not to answer	− 0.141 (− 0.205, − 0.077)***	− 12.4 (− 18.2, − 6.6)***
*Household income (reference: higher)*		
Middle	− 0.020 (− 0.032, − 0.009)***	− 2.3 (− 3.5, − 1.0)***
Lower	− 0.060 (− 0.072, − 0.048)***	− 5.8 (− 7.0, − 4.6)***
Prefer not to answer	− 0.035 (− 0.050, − 0.020)***	− 2.8 (− 4.2, − 1.3)***
*Ethnicity (reference: White)*		
Non-White	− 0.016 (− 0.051, 0.020)	− 1.9 (− 5.4, 1.6)
Prefer not to answer	− 0.058 (− 0.123, 0.007)	− 2.6 (− 7.6, 2.5)
*Caring (reference: none)*		
Caring < 20 h/week	0.001 (− 0.011, 0.014)	0.0 (− 1.3, 1.2)
Caring ≥ 20 h/week	− 0.053 (− 0.074, − 0.033)***	− 4.5 (− 6.2, − 2.8)***
Prefer not to answer	− 0.064 (− 0.113, − 0.016)**	− 6.9 (− 10.9, − 2.9)***
*Dependent children (reference: no)*		
Yes	0.017 (0.005, 0.030)**	1.0 (− 0.2, 2.2)
Prefer not to answer	− 0.154 (− 0.277, − 0.031)*	− 2.9 (− 12.4, 6.6)
Observations	7,747	7,747
R^2^	0.310	0.198

Models use heteroskedasticity-robust standard errors and adjust for recruitment-panel fixed effects, which are not shown. Models were restricted to respondents classified as male or female (n = 7,747) to align with the prespecified analytic base and permit direct comparison with the weighted analyses; 29 respondents recorded as “Other” gender were therefore excluded. Weighted estimates are reported in Table S8. Employed includes full-time and part-time employment or self-employment; unemployed or otherwise inactive includes respondents who were job-seeking, carers, looking after the home or family, students, or in another employment category

The EQ-5D-5​L index was calculated using the Rowen et al. [27] UK value set. Table S7 reports a Tobit sensitivity analysis censored at 1. EQ VAS ranges from 0 to 100. CI confidence interval. Values are rounded; significance symbols are based on unrounded p values. *p < 0.05; **p < 0.01; ***p < 0.001

Female sex and educational attainment below degree level were each associated with a slightly lower index, with coefficients of − 0.014 and − 0.011, respectively, but neither was associated with a lower EQ VAS. The models explained 31.0% of the variance in the EQ-5D-5​L index and 19.8% of the variance in EQ VAS.

### Robustness

The headline reference values were broadly robust to the analytical choices examined (Table S6). Weighting changed the overall mean index only marginally, from 0.820 to 0.827, and the mean EQ VAS from 72.6 to 73.1. In the sensitivity analyses using trimmed weights, an alternative four-band age-weighting scheme and the two definitions of rapid completion, the mean index ranged from 0.827 to 0.828 and the mean EQ VAS ranged from 73.0 to 73.2.

Estimates from the largest recruitment panel (n = 7,339) were similar to the base-case estimates. The two smaller panels, comprising 274 and 134 respondents, produced more variable estimates with wider confidence intervals. After standardisation, their mean index values were 0.813 and 0.880, respectively, compared with 0.828 in the largest panel (Table S6). The overall pattern of index associations was similar in the Tobit sensitivity analysis (Table S7), and the weighted ordinary least squares models produced broadly comparable findings for both outcomes (Table S8). In post hoc checks, the concentration of anxiety/depression problems among younger adults was unchanged within the largest recruitment panel and after excluding rapid completions, and the EQ-HWB-S anxiety and sadness/depression items and the ONS-4 anxiety item showed the same age pattern, each correlating strongly with the EQ-5D-5​L anxiety/depression dimension (Spearman ρ 0.62–0.75; Table S9).

## Discussion

This study provides contemporary EQ-5D-5​L reference values for adults in Scotland, derived from a large 2022 survey and, to our knowledge, scored for the first time using the recently published United Kingdom value set [27]. The overall weighted mean index was 0.827, the mean EQ VAS was 73.1 and approximately one-fifth of respondents reported full health. Problems were most prevalent in the pain/discomfort and anxiety/depression dimensions. Problems with mobility, usual activities and pain/discomfort generally increased with age, whereas anxiety/depression problems were most prevalent among younger adults, particularly young women. Women reported more problems than men across most age bands and dimensions. HRQoL was also markedly poorer among respondents classified as long-term sick or disabled and was lower among those with lower incomes or substantial caring responsibilities. Beyond confirming familiar age and sex patterns, the study provides Scotland-specific benchmarks scored with the new United Kingdom value set adopted by the National Institute for Health and Care Excellence for adult reference-case analyses, reference values by dimension and sociodemographic subgroup, and evidence that anxiety/depression problems were considerably more common, particularly among younger adults, than in earlier English EQ-5D-5​L data.

The age and sex patterns were broadly consistent with EQ-5D reference studies elsewhere. In the United States and Norway, problems in the physical dimensions generally became more prevalent with age, whereas anxiety/depression problems were more common among younger adults. Women also reported poorer health than men across most dimensions in the Norwegian study [12, 13]. A cross-country analysis of EQ-5D-3​L data similarly found poorer self-reported health with increasing age and among women, with mean EQ VAS scores ranging from 70.4 to 83.3 across 20 countries [9]. Because the EQ VAS is reported directly and does not depend on the value set, it is more comparable across studies, although sampling and administration differences remain important. The Scottish mean EQ VAS of 73.1 falls within this range [9].

Despite the increase in physical problems with age, the mean EQ-5D-5​L index did not decline monotonically across age groups. Lower anxiety/depression prevalence among older adults may partly offset increases in physical problems within the index. This highlights the importance of reporting dimension-level results alongside index values rather than relying on a single summary measure. Comparison with England is particularly relevant because English EQ-5D-5​L general-population data provide the closest available national comparator within the United Kingdom. Several patterns were similar: pain/discomfort was the most commonly affected dimension, problems in the physical dimensions generally increased with age, and women tended to report poorer health than men [11]. However, the concentration of anxiety/depression problems among younger adults observed in Scotland was not apparent in the English data [11]. That study analysed 996 adults interviewed face-to-face in 2012–2013, of whom 24.0% reported any anxiety/depression problems, compared with 53.9% in our weighted sample. The surveys were a decade apart, spanning the COVID-19 pandemic, and differed in mode of administration; online samples have reported lower utility values than matched face-to-face samples [12]. Differences in survey period, recruitment and administration mode may have contributed to the contrast, although their individual effects cannot be determined. The pattern in our data was also robust to the post hoc checks (Table S9).

Direct comparison of index values is difficult because the English study used the van Hout et al. crosswalk to the United Kingdom EQ-5D-3​L value set [11, 25], whereas the present study used the newly published, directly elicited United Kingdom EQ-5D-5​L value set [27]. Differences in sampling and survey administration further limit comparability. Nationally representative English EQ-5D-5​L reference values from the Health Survey for England 2017, scored using the same United Kingdom value set, are reported elsewhere [34]. Harmonised comparisons with these values are needed to distinguish genuine population variation from differences in sampling and survey administration.

Subgroup and multivariable analyses showed substantial variation in HRQoL beyond age and sex. Respondents classified as long-term sick or disabled had markedly lower EQ-5D-5​L index and EQ VAS scores than those in employment. This association should be interpreted cautiously because the employment category itself incorporates information about health and disability. Lower household income and providing at least 20 h of unpaid care per week were also associated with poorer outcomes after adjustment. These findings suggest that age- and sex-specific reference values alone may not capture important socioeconomic and caregiving-related heterogeneity. However, the cross-sectional design precludes causal interpretation, and residual confounding is likely. Nearly one in five respondents declined to report household income, as is common in surveys. Retaining them as a separate category avoided excluding a group with a somewhat lower mean index, whose adjusted index lay between those of the middle and lower income categories (Table 6).

The overall mean index differed by only 0.008 between the Rowen et al. [27] United Kingdom value set and the Devlin et al. [26] England value set, suggesting that the population mean was relatively insensitive to the choice between these value sets. However, the corresponding medians differed by 0.031, and the distributions differed in shape (Figure S1), indicating that similar means may conceal differences in the distribution of index values. Researchers should therefore report the value set used and avoid assuming that distributional summaries calculated using different scoring algorithms are interchangeable.

The headline estimates were stable under alternative weighting specifications and after excluding rapid completions. Estimates from the largest recruitment panel were also similar to the base-case estimates, whereas those from the two smaller panels were more variable and less precise. These differences may reflect sampling variability, panel composition or panel-specific recruitment processes, but the small panel sizes prevent these explanations from being distinguished.

The study has several strengths. It included 7,776 adults with complete EQ-5D-5​L profiles and EQ VAS data and followed a prespecified statistical analysis plan. Weighted and unweighted estimates were reported with confidence intervals, and robustness was assessed using alternative weighting specifications, completion-time exclusions, panel-standardised estimates, an alternative value set and alternative regression models. Reporting age- and sex-specific values, dimension-level problems, common health states and sociodemographic subgroups provides more comprehensive reference information than an overall population mean alone.

The principal limitation is the sampling design. Participants were recruited through commercial online panels rather than probability sampling. The achieved sample over-represented ​​​adults aged 45–74 years and women, under-represented​ adults aged 18–44 years and those aged 75 years or older, and included few respondents from minority ethnic groups. More than one-third of respondents had a university degree, although no population benchmark for education was applied. Post-stratification corrected the joint age-by-sex distribution but could not address selection within these cells or imbalances in education, deprivation and other measured or unmeasured characteristics. Although weighting had little effect on the headline estimates, residual selection bias cannot be excluded. A United States study found that participants recruited online reported lower utility values than a matched face-to-face sample [12], but its direction and magnitude here remain uncertain. We therefore describe the estimates as general-population reference values rather than nationally representative norms [32].

The absence of sufficiently detailed geographical information also prevented adjustment for the Scottish Index of Multiple Deprivation, health board or urban–rural classification. This is important given the substantial socioeconomic inequalities in health within Scotland [30]. Income and employment status cannot fully capture area-level deprivation or geographical variation. The survey also did not collect information on diagnosed or long-term health conditions, so reference values could not be described or stratified by health condition; employment status was the only available indicator of long-term sickness or disability. Other limitations include the cross-sectional design, reliance on self-reported data and the exclusion of 29 respondents recorded as “other” gender from weighted and multivariable analyses because no corresponding population target was available. The regression models identify associations rather than causal effects, and some explanatory categories, particularly long-term sickness or disability, may overlap conceptually with the outcomes being modelled.

These reference values provide age- and sex-specific benchmarks for interpreting patient-reported outcomes and assessing how the HRQoL of patient groups differs from that of the general population. They may also inform expected or baseline utility values in cost-effectiveness models, health technology assessment, burden-of-disease studies, clinical audits, disease registries and clinical trials conducted in Scotland [9, 10]. The dimension-level results may be particularly useful for identifying whether differences are concentrated in physical or mental health domains. The Scotland-specific estimates should complement rather than replace United Kingdom-wide reference values.

Future research should derive Scottish reference values using probability-based sampling and incorporate area-level deprivation, health-board and urban–rural characteristics. Larger samples of young adults, minority ethnic groups and people outside binary genœder categories would support more precise subgroup estimates. Repeated surveys are also needed to assess changes in population HRQoL over time, while harmonised comparisons across the constituent countries of the United Kingdom would help distinguish population differences from methodological variation.

## Conclusion

We provide contemporary age- and sex-specific EQ-5D-5​L reference values for adults in Scotland, derived from a large survey conducted in 2022 and calculated using the recently published United Kingdom value set. The estimates were broadly robust to the analytical choices examined and were consistent with the wider international EQ-5D literature. Because participants were recruited through non-probability online panels, the estimates should be regarded as general-population reference values rather than nationally representative norms. Subject to this limitation, they provide a contemporary benchmark for interpreting patient-reported outcomes, informing economic evaluation and health technology assessment, and assessing the burden of disease in Scottish research and practice.

## Supplementary Information

Below is the link to the electronic supplementary material.

Supplementary Material 1

## Data Availability

The SIPHER-HWMIC dataset analysed in this study is available from the UK Data Service (study number 9458; https://doi.org/10.5255/UKDA-SN-9458-1) under its End User Licence. No new datasets were generated.

## References

[1] Devlin, N. J., & Brooks, R. (2017). EQ-5D and the EuroQol group: Past, present and future. *Applied Health Economics and Health Policy, **15*(2), 127–137. 10.1007/s40258-017-0310-5 PMC5343080 28194657

[2] The EuroQol Group. (1990). EuroQol–a new facility for the measurement of health-related quality of life. *Health Policy, **16*(3), 199–208. 10.1016/0168-8510(90)90421-9 10109801

[3] Herdman, M., Gudex, C., Lloyd, A., Janssen, M., Kind, P., Parkin, D., et al. (2011). Development and preliminary testing of the new five-level version of EQ-5D (EQ-5D-5L). *Quality of Life Research, **20*(10), 1727–1736. 10.1007/s11136-011-9903-x PMC3220807 21479777

[4] Buchholz, I., Janssen, M. F., Kohlmann, T., & Feng, Y. S. (2018). A systematic review of studies comparing the measurement properties of the three-level and five-level versions of the EQ-5D. *PharmacoEconomics, **36*(6), 645–661. 10.1007/s40273-018-0642-5 PMC5954044 29572719

[5] Feng, Y. S., Kohlmann, T., Janssen, M. F., & Buchholz, I. (2021). Psychometric properties of the EQ-5D-5L: A systematic review of the literature. *Quality of Life Research, **30*(3), 647–673. 10.1007/s11136-020-02688-y PMC7952346 33284428

[6] Cheng, L. J., Pan, T., Chen, L. A., Cheng, J. Y., Mulhern, B., Devlin, N., et al. (2024). The ceiling effects of EQ-5D-3L and 5L in general population health surveys: A systematic review and meta-analysis. *Value in Health, **27*(7), 986–997. 10.1016/j.jval.2024.02.018 38467187

[7] Craig, B. M., Pickard, A. S., & Lubetkin, E. I. (2014). Health problems are more common, but less severe when measured using newer EQ-5D versions. *Journal of Clinical Epidemiology, **67*(1), 93–99. 10.1016/j.jclinepi.2013.07.011 PMC4091801 24075597

[8] Janssen, M. F., Bonsel, G. J., & Luo, N. (2018). Is EQ-5D-5L better than EQ-5D-3L? A head-to-head comparison of descriptive systems and value sets from seven countries. *PharmacoEconomics, **36*(6), 675–697. 10.1007/s40273-018-0623-8 PMC5954015 29470821

[9] Janssen, M. F., Szende, A., Cabases, J., Ramos-Goñi, J. M., Vilagut, G., & König, H. H. (2019). Population norms for the EQ-5D-3L: A cross-country analysis of population surveys for 20 countries. *The European Journal of Health Economics, **20*(2), 205–216. 10.1007/s10198-018-0955-5 PMC6438939 29445941

[10] Szende, A., Janssen, B., & Cabases, J. (Eds.). (2014). Self-reported population health: An international perspective based on EQ-5D. Springer. 29787044

[11] Feng, Y., Devlin, N., & Herdman, M. (2015). Assessing the health of the general population in England: How do the three- and five-level versions of EQ-5D compare? *Health and Quality of Life Outcomes, **13*, Article 171. 10.1186/s12955-015-0356-8 PMC4618130 26489956

[12] Jiang, R., Janssen, M. F. B., & Pickard, A. S. (2021). US population norms for the EQ-5D-5L and comparison of norms from face-to-face and online samples. *Quality of Life Research, **30*(3), 803–816. 10.1007/s11136-020-02650-y PMC7952367 33025373

[13] Garratt, A. M., Hansen, T. M., Augestad, L. A., Rand, K., & Stavem, K. (2022). Norwegian population norms for the EQ-5D-5L: Results from a general population survey. *Quality of Life Research, **31*(2), 517–526. 10.1007/s11136-021-02938-7 PMC8284681 34272631

[14] Grochtdreis, T., Dams, J., König, H. H., & Konnopka, A. (2019). Health-related quality of life measured with the EQ-5D-5L: Estimation of normative index values based on a representative German population sample and value set. *The European Journal of Health Economics, **20*(6), 933–944. 10.1007/s10198-019-01054-1 31030292

[15] Marten, O., & Greiner, W. (2021). EQ-5D-5L reference values for the German general elderly population. *Health and Quality of Life Outcomes, **19*(1), 76. 10.1186/s12955-021-01719-7 PMC7937199 33676523

[16] Meregaglia, M., Malandrini, F., Finch, A. P., Ciani, O., & Jommi, C. (2023). EQ-5D-5L population norms for Italy. *Applied Health Economics and Health Policy, **21*(2), 289–303. 10.1007/s40258-022-00772-7 PMC9702834 36434410

[17] Nikl, A., Janssen, M. F., Jenei, B., Brodszky, V., & Rencz, F. (2024). Population norms for the EQ-5D-5L, PROPr and SF-6D in Hungary. *PharmacoEconomics, **42*(5), 583–603. 10.1007/s40273-024-01360-4 PMC11039522 38416366

[18] Hobbins, A., Barry, L., Kelleher, D., & O’Neill, C. (2018). The health of the residents of Ireland: Population norms for Ireland based on the EQ-5D-5L descriptive system - A cross sectional study. *HRB Open Research, **1*, 22. 10.12688/hrbopenres.12848.1 PMC6973536 32002510

[19] Golicki, D., & Niewada, M. (2017). EQ-5D-5L Polish population norms. *Archives of Medical Science, **13*(1), 191–200. 10.5114/aoms.2015.52126 PMC5206353 28144271

[20] Hernandez, G., Garin, O., Pardo, Y., Vilagut, G., Pont, À., Suárez, M., et al. (2018). Validity of the EQ-5D-5L and reference norms for the Spanish population. *Quality of Life Research, **27*(9), 2337–2348. 10.1007/s11136-018-1877-5 29767329

[21] Encheva, M., Djambazov, S., Vekov, T., & Golicki, D. (2020). EQ-5D-5L Bulgarian population norms. ​The *European Journal of Health Economics, **21*(8), 1169–1178. 10.1007/s10198-020-01225-5 32813084

[22] Hołownia-Voloskova, M., Tarbastaev, A., & Golicki, D. (2021). Population norms of health-related quality of life in Moscow, Russia: The EQ-5D-5L-based survey. *Quality of Life Research, **30*(3), 831–840. 10.1007/s11136-020-02705-0 PMC7952340 33237551

[23] Vasan​ Thakumar, A., Li, X., Shafie, A. A., & Cheng, L. J. (2026). Population norms for the EQ-5D-3L and EQ-5D-5L in Peninsular Malaysia. *Quality of Life Research, **35*(5), 122. 10.1007/s11136-026-04231-x PMC13043599 41920393

[24] Xiamusiya, A., Lee, J. J., Shou, Y., Hao, S., Subramaniam, M., Cheng, L. J., et al. (2026). EQ-5D-5L population norms for Singapore: A household survey-based analysis. *Quality of Life Research, **35*(7), 181. 10.1007/s11136-026-04272-2 PMC13242447 42249989

[25] van Hout, B., Janssen, M. F., Feng, Y. S., Kohlmann, T., Busschbach, J., Golicki, D., et al. (2012). Interim scoring for the EQ-5D-5L: Mapping the EQ-5D-5L to EQ-5D-3L value sets. *Value in Health, **15*(5), 708–715. 10.1016/j.jval.2012.02.008 22867780

[26] Devlin, N. J., Shah, K. K., Feng, Y., Mulhern, B., & van Hout, B. (2018). Valuing health-related quality of life: An EQ-5D-5L value set for England. *Health Economics, **27*(1), 7–22. 10.1002/hec.3564 PMC6680214 28833869

[27] Rowen, D., Mukuria, C., Bray, N., Carlton, J., Longworth, L., Meads, D., et al. (2026). A United Kingdom Value Set for the EQ-5D-5L. *Value in Health​​​, **29*(5), 858–869. 10.1016/j.jval.2026.03.008 41887403

[28] National Institute for Health and Care Excellence. (2026). *Interim methods statement: Implementing the EQ-5D-5L value set* (PMG51). https://www.nice.org.uk/process/pmg51

[29] National Records of Scotland. (2025). *Life Expectancy in Scotland, 2022–2024*. https://nrscotland.gov.uk/publications/life-expectancy-in-scotland-2022-2024

[30] Scottish Government. (2025). *The Scottish health survey 2024 - Volume 1: Main report*.

[31] Wickramasekera, N., & Tsuchiya, A. (2025). A large scale population survey of health and wellbeing to allow comparisons between outcome measures: The SIPHER-HWMIC dataset. *Social Indicators Research, **180*(3), 1265–1290.

[32] Cornesse, C., Blom, A. G., Dutwin, D., Krosnick, J. A., De Leeuw, E. D., Legleye, S., et al. (2020). A review of conceptual approaches and empirical evidence on probability and nonprobability sample survey research. *Journal of Survey Statistics and Methodology, **8*(1), 4–36.

[33] National Records of Scotland. (2024). *Mid-2022 Population Estimates*. https://www.nrscotland.gov.uk/publications/mid-2022-population-estimates/

[34] Cheng, L. J., Li, X., & Vasan Thakumar, A. (2026). EQ-5D population norms for England: Comparing the UK and England EQ-5D-5L value sets, the former NICE reference-case mapping and the crosswalk. Quality of Life Research, 35, Article 319. 10.1007/s11136-026-04417-3 PMC13743634 42993413

