# Supplementary material for: Contemporary EQ-5D-5​L reference values for adults in Scotland: a secondary analysis of the SIPHER-HWMIC survey
Source: Qual Life Res. 2026 Nov 12;35(12):332. doi: 10.1007/s11136-026-04433-3 (PMC13759597; doi:10.1007/s11136-026-04433-3)
Supplement: Supplementary file 1 [file 11136_2026_4433_MOESM1_ESM.docx]

# Supplementary information

**Table S1.** EQ VAS, EQ-5D-5L index and full health by sociodemographic subgroup

**Panel A. Unweighted estimates**

| **Characteristic** | **n** | **EQ VAS mean (SD)** | **EQ-5D-5L index mean (SD)** | **Full health, %** |
| --- | --- | --- | --- | --- |
| **Highest educational qualification** | | | | |
| No formal qualification | 520 | 67.7 (23.7) | 0.752 (0.284) | 14.0 |
| GCSE or equivalent | 1,297 | 70.9 (21.8) | 0.799 (0.249) | 18.5 |
| A-level or equivalent | 1,491 | 72.6 (20.4) | 0.820 (0.236) | 20.5 |
| University degree (undergraduate or postgraduate) | 2,790 | 74.5 (18.9) | 0.852 (0.213) | 22.5 |
| Other qualification | 1,540 | 72.7 (21.2) | 0.811 (0.257) | 18.8 |
| Prefer not to answer | 138 | 64.5 (25.2) | 0.722 (0.356) | 15.2 |
| **Employment status** | | | | |
| Full-time (employed/self-employed) | 2,944 | 76.5 (16.6) | 0.884 (0.147) | 23.8 |
| Part-time (employed/self-employed) | 1,108 | 75.6 (18.0) | 0.866 (0.162) | 20.5 |
| Job-seeking | 112 | 68.4 (22.2) | 0.824 (0.181) | 11.6 |
| Long-term sick or disabled | 563 | 42.0 (21.5) | 0.363 (0.363) | 0.2 |
| Carer | 141 | 66.8 (22.9) | 0.762 (0.254) | 9.9 |
| Looking after home/family | 183 | 74.4 (19.1) | 0.851 (0.171) | 19.7 |
| Retired | 2,413 | 74.1 (20.0) | 0.832 (0.224) | 21.2 |
| Student | 132 | 76.1 (18.0) | 0.834 (0.166) | 18.9 |
| Other | 121 | 68.3 (23.3) | 0.754 (0.289) | 16.5 |
| Prefer not to answer | 59 | 62.1 (22.3) | 0.709 (0.244) | 11.9 |
| **Disposable household income after housing costs (monthly)** | | | | |
| ≤ £690 | 2,014 | 67.0 (22.8) | 0.749 (0.295) | 13.8 |
| £691–£1,040 | 1,187 | 72.8 (19.8) | 0.829 (0.225) | 19.4 |
| £1,041–£1,380 | 796 | 74.1 (19.5) | 0.845 (0.203) | 20.7 |
| £1,381–£1,730 | 609 | 75.6 (18.5) | 0.859 (0.187) | 21.8 |
| £1,731–£2,080 | 590 | 76.6 (17.6) | 0.881 (0.173) | 24.2 |
| > £2,080 | 1,131 | 78.5 (16.5) | 0.896 (0.155) | 30.7 |
| Prefer not to answer | 1,449 | 71.7 (22.0) | 0.796 (0.265) | 18.0 |
| **Total personal income before deductions, including benefits (annual)** | | | | |
| No income | 407 | 65.5 (24.4) | 0.699 (0.323) | 12.0 |
| ≤ £10,800 | 1,206 | 64.9 (23.5) | 0.724 (0.303) | 12.4 |
| £10,801–£14,400 | 907 | 70.9 (21.6) | 0.795 (0.270) | 17.6 |
| £14,401–£19,200 | 792 | 74.2 (19.7) | 0.837 (0.212) | 19.3 |
| £19,201–£26,400 | 1,142 | 75.9 (17.7) | 0.868 (0.165) | 20.9 |
| £26,401–£37,200 | 949 | 77.0 (16.1) | 0.888 (0.147) | 24.0 |
| £37,201–£52,800 | 655 | 77.3 (16.8) | 0.897 (0.149) | 30.2 |
| £52,801–£74,000 | 240 | 78.8 (14.6) | 0.906 (0.159) | 32.5 |
| > £74,000 | 148 | 80.9 (12.9) | 0.920 (0.115) | 37.2 |
| Prefer not to answer | 1,330 | 71.4 (22.4) | 0.796 (0.268) | 18.6 |
| **Ethnicity** | | | | |
| White | 7,603 | 72.6 (20.7) | 0.820 (0.242) | 20.1 |
| Black, Black British, Caribbean or Africanᶜ | 16 | — | — | — |
| Asian/Asian British | 37 | 74.8 (15.5) | 0.860 (0.132) | 21.6 |
| Mixed or multiple ethnic groups | 39 | 67.4 (22.9) | 0.810 (0.186) | 17.9 |
| Other ethnic groupᶜ | 12 | — | — | — |
| Prefer not to answer | 69 | 68.7 (23.2) | 0.747 (0.342) | 15.9 |
| **Responsibility for dependent children aged <18 years** | | | | |
| Yes | 1,377 | 74.0 (18.6) | 0.855 (0.196) | 23.0 |
| No | 6,376 | 72.3 (21.1) | 0.813 (0.250) | 19.4 |
| Prefer not to answerᶜ | 23 | — | — | — |
| **Caring responsibilities** | | | | |
| No | 6,048 | 73.2 (20.5) | 0.828 (0.239) | 21.4 |
| Yes, ≤9 h/week | 716 | 74.5 (18.7) | 0.846 (0.191) | 19.7 |
| Yes, 10–19 h/week | 222 | 72.6 (20.5) | 0.819 (0.219) | 16.2 |
| Yes, 20–34 h/week | 133 | 70.0 (19.0) | 0.771 (0.256) | 9.8 |
| Yes, 35–49 h/week | 146 | 64.5 (23.4) | 0.721 (0.306) | 13.7 |
| Yes, ≥50 h/week | 396 | 65.9 (22.6) | 0.740 (0.291) | 10.4 |
| Prefer not to answer | 115 | 62.1 (24.4) | 0.696 (0.323) | 9.6 |
| **Household size** | | | | |
| Living alone | 1,575 | 67.9 (23.0) | 0.768 (0.283) | 15.6 |
| 2 people | 3,858 | 73.6 (20.1) | 0.829 (0.235) | 20.4 |
| 3 people | 1,247 | 73.4 (20.3) | 0.831 (0.225) | 22.5 |
| 4 people | 819 | 75.0 (18.4) | 0.858 (0.198) | 23.1 |
| 5 or more people | 251 | 74.0 (19.6) | 0.831 (0.241) | 20.7 |
| Prefer not to answerᶜ | 26 | — | — | — |

Panel A includes all respondents in each subgroup. ᶜ Outcomes are not reported for subgroups with n < 30 because estimates would be unstable; the subgroup n is retained for transparency. SD standard deviation.

**Panel B. Estimates weighted to the National Records of Scotland mid-2022 age-band-by-sex distribution**

| **Characteristic** | **n** | **EQ VAS mean (95% CI)** | **EQ-5D-5L index mean (95% CI)** | **Full health, % (95% CI)** |
| --- | --- | --- | --- | --- |
| **Highest educational qualification** | | | | |
| No formal qualification | 520 | 67.9 (65.5, 70.2) | 0.753 (0.725, 0.780) | 13.7 (10.7, 17.4) |
| GCSE or equivalent | 1,293 | 70.9 (69.5, 72.3) | 0.801 (0.785, 0.817) | 19.1 (16.6, 21.9) |
| A-level or equivalent | 1,483 | 72.7 (71.4, 74.0) | 0.821 (0.807, 0.835) | 21.1 (18.5, 24.0) |
| University degree (undergraduate or postgraduate) | 2,776 | 75.4 (74.6, 76.1) | 0.864 (0.857, 0.871) | 23.6 (21.7, 25.6) |
| Other qualification | 1,537 | 73.1 (71.9, 74.3) | 0.816 (0.801, 0.830) | 18.7 (16.5, 21.2) |
| Prefer not to answer | 138 | 66.0 (61.6, 70.5) | 0.745 (0.683, 0.807) | 14.2 (8.8, 22.0) |
| **Employment status** | | | | |
| Full-time (employed/self-employed) | 2,934 | 76.7 (76.0, 77.4) | 0.889 (0.883, 0.895) | 25.4 (23.6, 27.4) |
| Part-time (employed/self-employed) | 1,103 | 75.1 (73.8, 76.5) | 0.862 (0.850, 0.873) | 20.7 (17.9, 23.8) |
| Job-seeking | 111 | 67.7 (60.7, 74.7) | 0.800 (0.739, 0.862) | 10.7 (5.7, 18.9) |
| Long-term sick or disabled | 558 | 43.0 (41.0, 45.1) | 0.389 (0.355, 0.423) | 0.5 (0.1, 3.2) |
| Carer | 141 | 65.4 (61.1, 69.7) | 0.746 (0.699, 0.794) | 7.7 (4.5, 12.9) |
| Looking after home/family | 183 | 73.7 (70.8, 76.7) | 0.847 (0.820, 0.873) | 18.3 (13.3, 24.8) |
| Retired | 2,412 | 74.4 (73.5, 75.3) | 0.831 (0.821, 0.842) | 20.2 (18.4, 22.1) |
| Student | 126 | 77.5 (74.3, 80.8) | 0.843 (0.807, 0.880) | 22.6 (14.5, 33.4) |
| Other | 120 | 69.3 (64.4, 74.3) | 0.749 (0.689, 0.808) | 13.5 (8.1, 21.7) |
| Prefer not to answer | 59 | 64.2 (56.8, 71.6) | 0.730 (0.676, 0.784) | 10.6 (4.7, 22.1) |
| **Disposable household income after housing costs (monthly)** | | | | |
| ≤ £690 | 2,006 | 67.8 (66.7, 68.9) | 0.761 (0.747, 0.774) | 13.8 (12.1, 15.7) |
| £691–£1,040 | 1,183 | 73.1 (71.6, 74.5) | 0.836 (0.821, 0.851) | 21.3 (18.4, 24.6) |
| £1,041–£1,380 | 796 | 74.3 (72.7, 75.9) | 0.847 (0.831, 0.863) | 20.3 (17.1, 23.9) |
| £1,381–£1,730 | 608 | 76.2 (74.5, 77.8) | 0.865 (0.849, 0.880) | 24.4 (20.3, 28.9) |
| £1,731–£2,080 | 588 | 76.9 (75.4, 78.5) | 0.885 (0.871, 0.900) | 26.6 (22.5, 31.1) |
| > £2,080 | 1,128 | 78.5 (77.5, 79.6) | 0.901 (0.892, 0.910) | 31.2 (28.1, 34.6) |
| Prefer not to answer | 1,438 | 72.4 (71.1, 73.7) | 0.802 (0.787, 0.817) | 17.7 (15.4, 20.3) |
| **Total personal income before deductions, including benefits (annual)** | | | | |
| No income | 403 | 66.7 (63.9, 69.5) | 0.721 (0.688, 0.755) | 12.2 (8.4, 17.5) |
| ≤ £10,800 | 1,199 | 66.1 (64.6, 67.7) | 0.737 (0.719, 0.756) | 13.2 (10.9, 16.0) |
| £10,801–£14,400 | 906 | 70.8 (69.1, 72.5) | 0.793 (0.773, 0.814) | 17.3 (14.4, 20.7) |
| £14,401–£19,200 | 790 | 74.2 (72.4, 76.0) | 0.841 (0.824, 0.859) | 20.0 (16.6, 23.9) |
| £19,201–£26,400 | 1,137 | 75.6 (74.3, 76.8) | 0.867 (0.856, 0.877) | 20.4 (17.8, 23.3) |
| £26,401–£37,200 | 945 | 77.4 (76.3, 78.5) | 0.894 (0.884, 0.904) | 27.0 (23.7, 30.6) |
| £37,201–£52,800 | 655 | 77.7 (76.3, 79.0) | 0.904 (0.893, 0.914) | 31.4 (27.4, 35.7) |
| £52,801–£74,000 | 240 | 78.5 (76.6, 80.5) | 0.913 (0.895, 0.930) | 33.1 (26.8, 40.0) |
| > £74,000 | 148 | 80.8 (78.5, 83.1) | 0.925 (0.907, 0.944) | 39.7 (30.6, 49.5) |
| Prefer not to answer | 1,324 | 72.0 (70.7, 73.4) | 0.796 (0.779, 0.813) | 17.2 (15.0, 19.7) |
| **Ethnicity** | | | | |
| White | 7,575 | 73.2 (72.6, 73.7) | 0.828 (0.822, 0.834) | 20.7 (19.6, 21.8) |
| Black, Black British, Caribbean or Africanᶜ | 16 | — | — | — |
| Asian/Asian British | 37 | 75.2 (70.1, 80.3) | 0.862 (0.818, 0.905) | 18.2 (8.2, 35.7) |
| Mixed or multiple ethnic groups | 38 | 59.0 (45.6, 72.4) | 0.756 (0.612, 0.900) | 17.7 (7.6, 35.7) |
| Other ethnic groupᶜ | 12 | — | — | — |
| Prefer not to answer | 69 | 70.4 (65.1, 75.7) | 0.766 (0.688, 0.845) | 19.1 (10.1, 33.1) |
| **Responsibility for dependent children aged <18 years** | | | | |
| Yes | 1,374 | 74.1 (73.1, 75.1) | 0.860 (0.850, 0.871) | 24.4 (21.9, 27.0) |
| No | 6,350 | 72.9 (72.3, 73.5) | 0.821 (0.814, 0.827) | 19.8 (18.6, 21.1) |
| Prefer not to answerᶜ | 23 | — | — | — |
| **Caring responsibilities** | | | | |
| No | 6,023 | 73.8 (73.2, 74.4) | 0.837 (0.831, 0.843) | 22.2 (20.9, 23.6) |
| Yes, ≤9 h/week | 712 | 74.6 (73.0, 76.1) | 0.846 (0.829, 0.862) | 19.3 (16.2, 22.7) |
| Yes, 10–19 h/week | 222 | 70.3 (65.7, 74.9) | 0.796 (0.750, 0.842) | 14.0 (9.9, 19.3) |
| Yes, 20–34 h/week | 133 | 69.3 (65.5, 73.2) | 0.763 (0.715, 0.811) | 10.6 (6.0, 18.1) |
| Yes, 35–49 h/week | 146 | 64.2 (60.2, 68.2) | 0.727 (0.676, 0.778) | 12.4 (7.8, 19.2) |
| Yes, ≥50 h/week | 396 | 65.6 (62.8, 68.3) | 0.728 (0.693, 0.763) | 9.1 (6.6, 12.3) |
| Prefer not to answer | 115 | 65.7 (60.6, 70.7) | 0.716 (0.657, 0.776) | 10.2 (5.2, 19.1) |
| **Household size** | | | | |
| Living alone | 1,571 | 69.0 (67.7, 70.4) | 0.782 (0.767, 0.797) | 16.5 (14.3, 19.0) |
| 2 people | 3,841 | 73.9 (73.2, 74.6) | 0.834 (0.826, 0.842) | 20.7 (19.2, 22.3) |
| 3 people | 1,242 | 73.9 (72.6, 75.2) | 0.836 (0.822, 0.851) | 22.7 (20.1, 25.6) |
| 4 people | 817 | 74.6 (73.0, 76.2) | 0.858 (0.842, 0.874) | 24.6 (21.1, 28.5) |
| 5 or more people | 251 | 75.8 (73.1, 78.5) | 0.845 (0.816, 0.875) | 20.9 (15.1, 28.2) |
| Prefer not to answerᶜ | 25 | — | — | — |

Panel B includes respondents classified as male or female with a valid post-stratification weight. Means have linearised 95% confidence intervals; confidence intervals for full-health percentages are bounded on the percentage scale.

The EQ-5D-5L index uses the Rowen et al. (2026) UK value set. Full health is health state 11111. ᶜ Outcomes are not reported for subgroups with unweighted n < 30 because estimates would be unstable; the subgroup n is retained for transparency.

**Table S2.** Ten most frequently reported EQ-5D-5L health states, ranked by unweighted frequency

| **EQ-5D-5L state** | **n** | **Unweighted, %** | **Weighted, %** |
| --- | --- | --- | --- |
| 11111 | 1,557 | 20.0 | 20.7 |
| 11121 | 899 | 11.6 | 10.6 |
| 11112 | 638 | 8.2 | 9.6 |
| 11122 | 549 | 7.1 | 7.1 |
| 11113 | 224 | 2.9 | 3.3 |
| 11123 | 209 | 2.7 | 3.1 |
| 21121 | 207 | 2.7 | 2.3 |
| 21221 | 196 | 2.5 | 2.3 |
| 21222 | 161 | 2.1 | 1.8 |
| 11222 | 106 | 1.4 | 1.4 |

Unweighted base: n = 7,776. Weighted percentages use the male/female analytic base (n = 7,747) and National Records of Scotland mid-2022 age-band-by-sex post-stratification weights.

A health state is a five-digit profile in the order mobility, self-care, usual activities, pain/discomfort and anxiety/depression, with levels ranging from 1 to 5.

EQ-5D-5L index values of these states under the Rowen et al. (2026) United Kingdom and Devlin et al. (2018) England value sets, respectively: 11111, 1.000 and 1.000; 11121, 0.944 and 0.937; 11112, 0.959 and 0.922; 11122, 0.903 and 0.859; 11113, 0.874 and 0.896; 11123, 0.818 and 0.833; 21121, 0.912 and 0.879; 21221, 0.863 and 0.829; 21222, 0.822 and 0.751; 11222, 0.854 and 0.809.

In total, 594 of the 3,125 possible EQ-5D-5L health states (19.0%) were reported, 284 of them by a single respondent.

**Table S3.** Distribution of the EQ-5D-5L level sum score

| **Level sum score** | **n** | **Unweighted, %** | **Weighted, %** |
| --- | --- | --- | --- |
| 5 | 1,557 | 20.0 | 20.7 |
| 6 | 1,601 | 20.6 | 21.0 |
| 7 | 1,231 | 15.8 | 16.0 |
| 8 | 850 | 10.9 | 11.5 |
| 9 | 617 | 7.9 | 7.7 |
| 10 | 451 | 5.8 | 5.8 |
| 11 | 297 | 3.8 | 3.7 |
| 12 | 239 | 3.1 | 2.7 |
| 13 | 181 | 2.3 | 2.3 |
| 14 | 148 | 1.9 | 1.7 |
| 15 | 152 | 2.0 | 1.9 |
| 16 | 122 | 1.6 | 1.3 |
| 17 | 95 | 1.2 | 1.1 |
| 18 | 71 | 0.9 | 0.7 |
| 19 | 59 | 0.8 | 0.7 |
| 20 | 49 | 0.6 | 0.5 |
| 21 | 28 | 0.4 | 0.3 |
| 22 | 16 | 0.2 | 0.2 |
| 23 | 4 | 0.1 | <0.1 |
| 24 | 5 | 0.1 | 0.1 |
| 25 | 3 | <0.1 | <0.1 |

Mean level sum score: unweighted 8.22 (8.14, 8.29); weighted 8.06 (7.97, 8.14).

The level sum score is the sum of the five dimension levels and ranges from 5 to 25. Weighted percentages use the male/female analytic base.

**Table S4.** Number of EQ-5D-5L dimensions with problems

| **Dimensions with problems** | **n** | **Unweighted, %** | **Weighted, %** |
| --- | --- | --- | --- |
| 0 | 1,557 | 20.0 | 20.7 |
| 1 | 1,962 | 25.2 | 26.3 |
| 2 | 1,488 | 19.1 | 19.9 |
| 3 | 1,022 | 13.1 | 13.0 |
| 4 | 922 | 11.9 | 10.7 |
| 5 | 825 | 10.6 | 9.4 |

A dimension was counted as having problems when reported at level 2 or higher. Weighted percentages use the male/female analytic base.

**Table S5.** EQ-5D-5L index under alternative value sets

| **Value set** | **Analysis** | **n** | **Mean (SD)** | **95% CI** | **Median** | **Full health, %** |
| --- | --- | --- | --- | --- | --- | --- |
| Rowen 2026 (UK) | Unweighted | 7,776 | 0.820 (0.242) | (0.814, 0.825) | 0.903 | 20.0 |
| Rowen 2026 (UK) | Weighted | 7,747 | 0.827 (0.231) | (0.821, 0.833) | 0.903 | 20.7 |
| Devlin 2018 (England) | Unweighted | 7,776 | 0.811 (0.214) | (0.806, 0.816) | 0.859 | 20.0 |
| Devlin 2018 (England) | Weighted | 7,747 | 0.819 (0.204) | (0.814, 0.824) | 0.872 | 20.7 |

Full health is health state 11111 and therefore has the same prevalence under both value sets. CI confidence interval, SD standard deviation.

**Distribution of index values under each value set**

| **Value set** | **Analysis** | **n** | **Minimum** | **P10** | **P25** | **Median** | **P75** | **P90** | **Below 0, %** |
| --- | --- | --- | --- | --- | --- | --- | --- | --- | --- |
| Rowen 2026 (UK) | Unweighted | 7,776 | −0.567 | 0.522 | 0.796 | 0.903 | 0.959 | 1.000 | 2.2 |
| Rowen 2026 (UK) | Weighted | 7,747 | −0.567 | 0.550 | 0.808 | 0.903 | 0.959 | 1.000 | 1.7 |
| Devlin 2018 (England) | Unweighted | 7,776 | −0.285 | 0.542 | 0.751 | 0.859 | 0.937 | 1.000 | 1.2 |
| Devlin 2018 (England) | Weighted | 7,747 | −0.285 | 0.581 | 0.758 | 0.872 | 0.937 | 1.000 | 1.0 |

Weighted rows use the male/female analytic base (n = 7,747) and National Records of Scotland mid-2022 age-band-by-sex post-stratification weights. Values below 0 indicate health states valued as worse than dead. P10–P90 10th to 90th percentiles.

**Table S6.** Sensitivity analyses of headline reference values

| **Scenario** | **n** | **EQ-5D-5L index mean (95% CI)** | **EQ VAS mean (95% CI)** | **Full health, % (95% CI)** | **Any pain/discomfort, % (95% CI)** |
| --- | --- | --- | --- | --- | --- |
| 1 Base: weighted 7-band | 7,747 | 0.827 (0.821, 0.833) | 73.1 (72.6, 73.6) | 20.7 (19.6, 21.8) | 60.3 (58.9, 61.6) |
| 2 Unweighted | 7,776 | 0.820 (0.814, 0.825) | 72.6 (72.1, 73.0) | 20.0 (19.1, 20.9) | 64.0 (62.9, 65.1) |
| 3 Trimmed weights (1st/99th percentiles) | 7,747 | 0.827 (0.821, 0.832) | 73.0 (72.5, 73.5) | 20.5 (19.5, 21.6) | 60.9 (59.6, 62.2) |
| 4 Alternative four-band age weighting (18–34, 35–54, 55–74 and ≥75 years, by sex) | 7,747 | 0.828 (0.823, 0.834) | 73.1 (72.6, 73.6) | 20.9 (19.9, 22.0) | 60.5 (59.2, 61.7) |
| 5 Excluded completion time <6 min (a priori; reweighted) | 7,636 | 0.827 (0.821, 0.833) | 73.1 (72.6, 73.6) | 20.3 (19.2, 21.5) | 60.5 (59.1, 61.9) |
| 6 Excluded speeders (<median/3; reweighted) | 7,726 | 0.828 (0.822, 0.834) | 73.2 (72.7, 73.7) | 20.7 (19.6, 21.9) | 60.3 (58.9, 61.6) |
| 7a Panel: MRFGR (standardised) | 274 | 0.813 (0.782, 0.845) | 72.0 (69.4, 74.6) | 22.9 (18.0, 28.6) | 60.2 (53.9, 66.2) |
| 7b Panel: Prolific (standardised) | 134 | 0.880 (0.844, 0.917) | 75.7 (72.9, 78.6) | 31.0 (22.8, 40.6) | 43.0 (33.7, 53.0) |
| 7c Panel: STV (standardised) | 7,339 | 0.828 (0.822, 0.834) | 73.1 (72.6, 73.7) | 20.3 (19.2, 21.5) | 60.6 (59.2, 62.0) |

Each scenario changes one analytical decision relative to the weighted seven-band base case. Weighted scenarios were re-post-stratified within the scenario-specific sample; recruitment-panel estimates were each standardised to the National Records of Scotland age-band-by-sex distribution.

Confidence intervals for means use linearised standard errors; confidence intervals for binary outcomes are logit-transformed and bounded on the percentage scale.

**Table S7.** Sensitivity of EQ-5D-5L index associations to model choice: ordinary least squares and Tobit regression

| **Characteristic** | **OLS β (95% CI)** | **Tobit β (95% CI)** |
| --- | --- | --- |
| **Age band (reference: 45–54)** | | |
| 18–24 | 0.010 (−0.015, 0.036) | 0.009 (−0.021, 0.039) |
| 25–34 | 0.021 (0.006, 0.036)** | 0.028 (0.009, 0.048)** |
| 35–44 | 0.004 (−0.012, 0.020) | −0.003 (−0.023, 0.016) |
| 55–64 | 0.018 (0.002, 0.033)* | 0.021 (0.003, 0.039)* |
| 65–74 | −0.006 (−0.025, 0.014) | −0.010 (−0.033, 0.014) |
| 75+ | 0.000 (−0.027, 0.026) | −0.012 (−0.043, 0.019) |
| **Sex (reference: male)** | | |
| Female | −0.014 (−0.023, −0.004)** | −0.017 (−0.028, −0.005)** |
| **Highest qualification (reference: degree)** | | |
| Below degree | −0.011 (−0.020, −0.002)* | −0.013 (−0.024, −0.002)* |
| Prefer not to answer | −0.045 (−0.093, 0.003) | −0.049 (−0.102, 0.005) |
| **Employment (reference: employed)** | | |
| Retired | −0.033 (−0.048, −0.018)*** | −0.032 (−0.050, −0.013)*** |
| Long-term sick or disabled | −0.489 (−0.519, −0.458)*** | −0.518 (−0.549, −0.487)*** |
| Unemployed or other inactive | −0.049 (−0.067, −0.031)*** | −0.058 (−0.079, −0.037)*** |
| Prefer not to answer | −0.141 (−0.205, −0.077)*** | −0.158 (−0.229, −0.088)*** |
| **Household income (reference: higher)** | | |
| Middle | −0.020 (−0.032, −0.009)*** | −0.036 (−0.052, −0.020)*** |
| Lower | −0.060 (−0.072, −0.048)*** | −0.085 (−0.101, −0.069)*** |
| Prefer not to answer | −0.035 (−0.050, −0.020)*** | −0.054 (−0.073, −0.035)*** |
| **Ethnicity (reference: White)** | | |
| Non-White | −0.016 (−0.051, 0.020) | −0.018 (−0.062, 0.026) |
| Prefer not to answer | −0.058 (−0.123, 0.007) | −0.065 (−0.137, 0.008) |
| **Caring (reference: none)** | | |
| Caring <20 h/week | 0.001 (−0.011, 0.014) | −0.003 (−0.019, 0.012) |
| Caring ≥20 h/week | −0.053 (−0.074, −0.033)*** | −0.068 (−0.090, −0.045)*** |
| Prefer not to answer | −0.064 (−0.113, −0.016)** | −0.079 (−0.130, −0.027)** |
| **Dependent children (reference: no)** | | |
| Yes | 0.017 (0.005, 0.030)** | 0.026 (0.010, 0.041)** |
| Prefer not to answer | −0.154 (−0.277, −0.031)* | −0.157 (−0.289, −0.026)* |
| Observations | 7,747 | 7,747 |

Both models use the same unweighted analytic sample and covariates as Table 6. The Tobit model is right-censored at an index of 1; 1,554 observations (20.1%) were censored. Tobit coefficients refer to the latent outcome and should not be interpreted as marginal changes in observed EQ-5D-5L index values. Employed includes full-time and part-time employment or self-employment; unemployed or otherwise inactive includes respondents who were job-seeking, carers, looking after the home or family, students, or in another employment category.

CI confidence interval. Values are rounded; significance symbols are based on unrounded p values. *p < 0.05; **p < 0.01; ***p < 0.001.

**Table S8.** Factors associated with the EQ-5D-5L index and EQ VAS: weighted multivariable ordinary least squares models

| **Characteristic** | **Weighted EQ-5D-5L index, β (95% CI)** | **Weighted EQ VAS, β (95% CI)** |
| --- | --- | --- |
| **Age band (reference: 45–54)** | | |
| 18–24 | 0.018 (−0.008, 0.045) | 4.6 (2.1, 7.1)*** |
| 25–34 | 0.021 (0.006, 0.036)** | 2.4 (0.8, 4.0)** |
| 35–44 | −0.002 (−0.018, 0.015) | −0.3 (−1.8, 1.3) |
| 55–64 | 0.015 (0.000, 0.030)* | 2.3 (0.9, 3.7)*** |
| 65–74 | −0.008 (−0.028, 0.012) | −0.1 (−2.0, 1.9) |
| 75+ | −0.001 (−0.029, 0.027) | 0.8 (−2.0, 3.7) |
| **Sex (reference: male)** | | |
| Female | −0.017 (−0.027, −0.006)** | 0.2 (−0.8, 1.1) |
| **Highest qualification (reference: degree)** | | |
| Below degree | −0.019 (−0.029, −0.009)*** | −1.0 (−2.0, 0.0)* |
| Prefer not to answer | −0.048 (−0.102, 0.005) | −5.4 (−9.5, −1.4)** |
| **Employment (reference: employed)** | | |
| Retired | −0.031 (−0.047, −0.014)*** | −0.7 (−2.6, 1.1) |
| Long-term sick or disabled | −0.465 (−0.499, −0.431)*** | −31.3 (−33.4, −29.1)*** |
| Unemployed or other inactive | −0.054 (−0.075, −0.033)*** | −3.8 (−5.9, −1.7)*** |
| Prefer not to answer | −0.123 (−0.179, −0.067)*** | −10.7 (−17.5, −3.9)** |
| **Household income (reference: higher)** | | |
| Middle | −0.022 (−0.034, −0.010)*** | −2.0 (−3.3, −0.6)** |
| Lower | −0.057 (−0.069, −0.046)*** | −5.3 (−6.7, −4.0)*** |
| Prefer not to answer | −0.032 (−0.049, −0.016)*** | −2.1 (−3.8, −0.5)* |
| **Ethnicity (reference: White)** | | |
| Non-White | −0.008 (−0.049, 0.034) | −2.9 (−7.7, 1.8) |
| Prefer not to answer | −0.050 (−0.114, 0.015) | −1.5 (−6.4, 3.5) |
| **Caring (reference: none)** | | |
| Caring <20 h/week | −0.008 (−0.022, 0.007) | −0.4 (−1.9, 1.0) |
| Caring ≥20 h/week | −0.062 (−0.085, −0.038)*** | −4.8 (−6.7, −2.9)*** |
| Prefer not to answer | −0.044 (−0.090, 0.002) | −2.9 (−7.2, 1.4) |
| **Dependent children (reference: no)** | | |
| Yes | 0.018 (0.005, 0.031)** | 0.9 (−0.4, 2.2) |
| Prefer not to answer | −0.179 (−0.340, −0.018)* | −11.5 (−28.6, 5.6) |
| Observations | 7,747 | 7,747 |
| R² | 0.299 | 0.189 |

Models use National Records of Scotland mid-2022 age-band-by-sex post-stratification weights, heteroskedasticity-robust standard errors and recruitment-panel fixed effects, which are not shown. The analytic sample and covariates are the same as in Table 6 (n = 7,747). Employed includes full-time and part-time employment or self-employment; unemployed or otherwise inactive includes respondents who were job-seeking, carers, looking after the home or family, students, or in another employment category.

CI confidence interval. Values are rounded; significance symbols are based on unrounded p values. *p < 0.05; **p < 0.01; ***p < 0.001.

**Table S9.** Post hoc checks of the age pattern of anxiety/depression problems

**Panel A. Any anxiety/depression problems (EQ-5D-5L levels 2–5), % (n)**

| **Age band** | **Full analytic sample** | **Largest panel (STV) only** | **Excluding completion <6 min** | **Excluding completion <median/3** |
| --- | --- | --- | --- | --- |
| **Males** | | | | |
| 18–24 | 65.7 (70) | 64.9 (57) | 66.2 (65) | 64.2 (67) |
| 25–34 | 60.4 (250) | 62.2 (217) | 61.0 (241) | 60.6 (249) |
| 35–44 | 61.6 (315) | 62.7 (271) | 61.7 (300) | 61.5 (314) |
| 45–54 | 50.5 (503) | 51.2 (461) | 50.9 (495) | 50.6 (502) |
| 55–64 | 45.1 (861) | 45.0 (831) | 45.1 (855) | 45.1 (859) |
| 65–74 | 35.5 (898) | 35.5 (866) | 35.6 (897) | 35.5 (898) |
| 75+ | 30.2 (308) | 31.2 (295) | 30.2 (308) | 30.2 (308) |
| **Females** | | | | |
| 18–24 | 79.9 (229) | 78.7 (202) | 80.5 (210) | 79.7 (222) |
| 25–34 | 66.1 (567) | 65.6 (538) | 66.8 (552) | 66.3 (564) |
| 35–44 | 69.1 (660) | 69.8 (625) | 69.6 (644) | 69.3 (657) |
| 45–54 | 58.4 (906) | 58.1 (871) | 58.6 (898) | 58.4 (906) |
| 55–64 | 51.8 (1,185) | 51.7 (1,144) | 52.0 (1,179) | 51.8 (1,185) |
| 65–74 | 43.0 (810) | 42.9 (787) | 43.1 (807) | 43.0 (810) |
| 75+ | 34.1 (185) | 33.3 (174) | 34.1 (185) | 34.1 (185) |

**Panel B. Other mental-health items in the same survey, mean (95% CI)**

| **Age band** | **n** | **EQ-HWB-S anxious (0–4)** | **EQ-HWB-S sad or depressed (0–4)** | **ONS-4 anxiety (0–10)** |
| --- | --- | --- | --- | --- |
| **Males** | | | | |
| 18–24 | 70 | 1.87 (1.57, 2.17) | 1.59 (1.29, 1.88) | 4.39 (3.67, 5.10) |
| 25–34 | 250 | 1.53 (1.38, 1.69) | 1.35 (1.20, 1.49) | 3.95 (3.60, 4.30) |
| 35–44 | 315 | 1.64 (1.50, 1.77) | 1.58 (1.45, 1.72) | 4.37 (4.03, 4.70) |
| 45–54 | 503 | 1.26 (1.16, 1.37) | 1.24 (1.13, 1.35) | 3.61 (3.35, 3.88) |
| 55–64 | 861 | 1.05 (0.97, 1.13) | 1.03 (0.95, 1.10) | 3.07 (2.86, 3.27) |
| 65–74 | 898 | 0.77 (0.71, 0.83) | 0.80 (0.73, 0.86) | 2.63 (2.44, 2.83) |
| 75+ | 308 | 0.66 (0.56, 0.76) | 0.68 (0.57, 0.79) | 2.45 (2.13, 2.78) |
| **Females** | | | | |
| 18–24 | 229 | 2.33 (2.17, 2.49) | 1.94 (1.77, 2.11) | 5.48 (5.11, 5.84) |
| 25–34 | 567 | 1.99 (1.89, 2.09) | 1.59 (1.49, 1.69) | 5.08 (4.84, 5.32) |
| 35–44 | 660 | 1.80 (1.71, 1.90) | 1.53 (1.44, 1.62) | 4.75 (4.53, 4.98) |
| 45–54 | 906 | 1.59 (1.51, 1.67) | 1.41 (1.33, 1.49) | 4.25 (4.04, 4.45) |
| 55–64 | 1,185 | 1.35 (1.29, 1.42) | 1.29 (1.22, 1.36) | 3.73 (3.55, 3.91) |
| 65–74 | 810 | 1.09 (1.02, 1.17) | 1.03 (0.95, 1.10) | 3.19 (2.97, 3.40) |
| 75+ | 185 | 0.85 (0.71, 0.99) | 0.79 (0.65, 0.93) | 3.26 (2.79, 3.73) |
| All respondents | 7,776 | 1.35 (1.32, 1.37) | 1.23 (1.20, 1.26) | 3.75 (3.68, 3.82) |
| Spearman ρ with EQ-5D-5L anxiety/depression | 7,776 | 0.75 | 0.75 | 0.62 |

Panel A shows unweighted percentages with cell n in parentheses, in the full analytic sample, in the largest recruitment panel only, and after excluding completion times under 6 minutes or under one-third of the median. Panel B shows unweighted means of the EQ-HWB-S items “Did you feel anxious” and “Did you feel sad or depressed” (last 7 days; 0 none of the time to 4 most or all of the time) and the ONS-4 item “How anxious did you feel yesterday” (0 not at all to 10 completely); higher scores indicate more anxiety or sadness. The last row gives Spearman correlations with the EQ-5D-5L anxiety/depression level.

Post hoc analysis in response to peer review. Age-band-by-sex estimates are weight-invariant, so unweighted estimates are shown; all respondents, including those recorded as “Other” gender, are included in the overall rows. CI confidence interval; EQ-HWB-S EQ Health and Wellbeing Short; ONS-4 Office for National Statistics personal well-being questions.

**Figure S1.** Distribution of EQ-5D-5L index values under the Rowen et al. (2026) United Kingdom and Devlin et al. (2018) England value sets. Histograms show the weighted percentage of respondents (n = 7,747) in bins of width 0.025, weighted to the National Records of Scotland mid-2022 age-by-sex distribution; dashed lines mark the medians. Index values cluster on a small number of frequently reported health states, so the medians differ more than the means.
